# Conformational Preference of ‘C^α^NN’ Short Peptide Motif towards Recognition of Anions

**DOI:** 10.1371/journal.pone.0057366

**Published:** 2013-03-13

**Authors:** Tridip Sheet, Subhrangshu Supakar, Raja Banerjee

**Affiliations:** Department of Bioinformatics, West Bengal University of Technology, Salt Lake, Kolkata, India; Instituto de Tecnologica Química e Biológica, UNL, Portugal

## Abstract

Among several ‘anion binding motifs’, the recently described ‘C^α^NN’ motif occurring in the loop regions preceding a helix, is conserved through evolution both in sequence and its conformation. To establish the significance of the conserved sequence and their intrinsic affinity for anions, a series of peptides containing the naturally occurring ‘C^α^NN’ motif at the N-terminus of a designed helix, have been modeled and studied in a context free system using computational techniques. Appearance of a single interacting site with negative binding free-energy for both the sulfate and phosphate ions, as evidenced in docking experiments, establishes that the ‘C^α^NN’ segment has an intrinsic affinity for anions. Molecular Dynamics (MD) simulation studies reveal that interaction with anion triggers a conformational switch from non-helical to helical state at the ‘C^α^NN’ segment, which extends the length of the anchoring-helix by one turn at the N-terminus. Computational experiments substantiate the significance of sequence/structural context and justify the conserved nature of the ‘C^α^NN’ sequence for anion recognition through “local” interaction.

## Introduction

Proteins, the ubiquitous biopolymers, in several occasions interact with anions through non-covalent interactions which modulate and stabilize their 3D-structures. In addition, such anion-protein interactions play crucial role in regulating various important biological processes [1]–[6] and sometimes participate in amending several biophysical features at the molecular level [7]–[10].

Analyses of multiple families of proteins with different folds have shown that specific sites, mainly comprised of the main-chain atoms of three to four amino acid residues, participate in the formation of a “functional surface” where anions usually reside and that the interaction is mediated by proper positioning of the polypeptide backbone [11]–[14]. Among the several recognized ‘anion binding motifs’ [*e.g.* ‘C^α^NN’ [13], ‘nests’ [14]–[16], ‘structural P-loop’ [17], ‘cup’ [18] along with motifs for recognizing adenine, adenine-containing nucleotides and its analogues [12], [19], [20]], the recently identified ‘C^α^NN’ motif [13], common to more than 100 fold-representative protein structures as observed in the FSSP database, has a specially characteristic feature. This motif consists of main chain atoms of three consecutive residues (C^α^_−1_N_0_, N_+1_), often present in the active sites of proteins and participates directly in several key regulating functions [1]–[3], [21].

This ‘C^α^NN’ motif, evolutionarily conserved in sequence (having GXX ‘sequence motif’) and conformation, normally occurs in a loop region preceding a helix (anchoring helix) [13], [22], [23]. Further, this motif, usually possessing a spatial geometry of a right-handed βαα or βαβ backbone conformation, upon interaction with anion endures an accompanying conformational change from a non-helical to a helical state at the ‘C^α^NN’ segment that extends the length of the anchoring-helix by one turn towards its N-terminus.

Fifty years ago, the chemistry Nobel laureate C.B. Anfinsen hypothesized that “*… the information ……. of the native secondary and tertiary structures* (of proteins) *is contained in the amino acid sequence itself*” [24]. As the ‘C^α^NN’ motif is found to be conserved in sequence and conformation through evolution, it would be worthy to know whether the information regarding its anion recognition is embedded in the local sequences and its conformational facet during anion recognition. Moreover, participation of the protein tertiary structure in affecting the N-terminal helix-extension upon anion binding has also to be corroborated. In a recent publication [25] using complementary spectroscopic techniques we have presented evidence for the interaction of a sulfate (SO_4_^2−^) ion with a ‘C^α^NN motif’ segment (Gly-Lys-Gln from protein 1MUG) in an 18-residue designed chimeric peptide sequence in a context free system as reported in protein crystal structures.

To ensure the significance of the conserved nature of the sequence pattern in the ‘C^α^NN’ motif as well as to characterize the nature of interaction of anion with this protein segment, whether ‘local’ or ‘global’; using computational and experimental approaches, we report here the interactions of both the sulfate (SO_4_^2−^) and phosphate (HPO_4_^2−^) ions (anions required by all cells for maintaining their normal function) [26], with several chimeric polypeptide sequences where the ‘C^α^NN’ anion binding structural motif containing residues have been appended at the N-terminus of these context free designed sequences. Molecular docking and molecular dynamics (MD) simulation studies have been employed to validate the nature of anion binding interaction along with its feasibility from the thermodynamic viewpoint and to monitor the detailed kinetic view of the accompanying conformational changes; while ESI-MS experiments are used to confirm the binding of anion(s) to the peptide. We describe here the conformational preference of the ‘C^α^NN’ motif during anion recognition which substantiate that the information regarding its anion recognition is embedded in the ‘local sequences’. This study would help in understanding the sequence/structural context of anion recognition in proteins as proposed [12], [13], [14] along with the relevance of the associated thermodynamic parameters in binding interaction.

## Results and Discussion

In order to rationalize the conformational preference of the ‘C^α^NN’ motif during its interactions with anion(s) [sulfate (SO_4_^2−^) and phosphate (HPO_4_^2−^) ions [11]–[13], [16]], a series of context free 18-residue and 5-residue chimeric polypeptide sequences containing the naturally occurring ‘C^α^NN’ anion binding structural motif have been designed. Interaction with the anion(s) is studied using different computational approaches and results are discussed below.

### I. Design of peptide sequences and structure building

A series of 18-residue chimeric polypeptides (CPS224Ac, CPS226, CPS228) have been designed where the ‘C^α^NN’ anion binding structural motif containing residues [*e.g.* Leu-Gly-Lys-Gln from protein DNA-glycosylase (1MUG, residues 107–110) for CPS224Ac; Gly-Ser-Ala-Lys from protein Cytochrome C (1YCC, residues 1–4) for CPS226; Leu-Gly-Gly-Leu from protein Activating enzymes of the ubiquitin-like proteins (1JW9, residues 39–42) for CPS228] [13] have been appended at the N-terminus of a designed context free model anchoring helix (ABGY) [27], [28] that contained the helicogenenic Aib residue [29], [30] (Table 1). Another series of 5-residue short sequences (SCPS224Ac, SCPS226, and SCPS228) have also been designed where the anchoring helix (ABGY), after the ‘C^α^NN’ motif containing residues, has been replaced by ‘Ala’ residue only (Table 1). For computational works the starting atomic coordinates of the peptides (CPS224Ac, CPS226, CPS228 and SCPS224Ac, SCPS226, SCPS228) have been built as follows:

**Table 1:** Details of designed peptide sequences used for computational work.

Peptide name	PDB entries^a^	‘C^α^NN’ motif segment^b^			Designed sequences of peptides used for computational study^c^
CPS 224Ac	1MUG (107–110)	Leu-Gly-Lys-Gln			Ac-Leu-Gly-Lys-Gln-Ala-Aib-Ala-Lys-Ala-Aib-Lys-Ala-Lys-Ala-Aib-Gly-Gly-Tyr-NH_2_
			φ	ψ	
		Leu	−89.1	87.2	
		Gly	130.6	126.7	
		Lys	−63.7	−45.1	
		Gln	−59.2	−46.7	
SCPS 224Ac	1MUG (107–110)	Leu-Gly-Lys-Gln			Ac-Leu-Gly-Lys-Gln-Ala-NH_2_
CPS 226	1YCC (1–4)	Gly-Ser-Ala-Lys			Ac-Gly-Ser-Ala-Lys-Ala-Aib-Ala-Lys-Ala-Aib-Lys-Ala-Lys-Ala-Aib-Gly-Gly-Tyr-NH_2_
			φ	ψ	
		Gly	−131.8	−146.2	
		Ser	−97.3	115.6	
		Ala	−67.7	−35.0	
		Lys	−68.1	−47.8	
SCPS 226	1YCC (1–4)	Gly-Ser-Ala-Lys			Ac-Gly-Ser-Ala-Lys-Ala-NH_2_
CPS 228	1JW9 (39–42)	Leu-Gly-Gly-Leu			Ac-Leu-Gly-Gly-Leu-Ala-Aib-Ala-Lys-Ala-Aib-Lys-Ala-Lys-Ala-Aib-Gly-Gly-Tyr-NH_2_
			φ	ψ	
		Leu	−112.9	46.7	
		Gly	−95.1	−139.5	
		Gly	−63.0	−56.2	
		Leu	−62.1	−42.1	
SCPS 228	1JW9 (39–42)	Leu-Gly-Gly-Leu			Ac-Leu-Gly-Gly-Leu-Ala-NH_2_

a PDB IDs from where the ‘C^α^NN’ motif segment residues are taken, residue number of PDB IDs are in parenthesis; the

b ‘C^α^NN’ motif segment is underlined;

c anchor helix (ABGY) sequence: -Ala-Aib-Ala-Lys-Ala-Aib-Lys-Ala-Lys-Ala-Aib-Gly-Gly-Tyr-NH_2_.

#### a. Experimental NMR structure

Translating the αN and NN nuclear Overhauser effect (nOe) cross-peak intensities, obtained from the interaction between CPS224Ac and the sulfate ion in the 2D-NMR (nuclear magnetic resonance) experiments [25], into suitable distance upper limits (NOE ∝ (1/r)^6^) [weak (w): 4.25 Å, medium (m): 3.5 Å, strong (s): 3.0 Å] and the ^3^J_Nα_ values of individual residues into the backbone dihedral angles (φ±10) using Karplus equation (^3^J_Nα_ = 6.4Cos^2^θ−1.4Cosθ+1.9 where, θ = |φ−60^0^|) [31], 100 structures are generated by the programme DYANA (Dynamics Algorithm for NMR Application) [32]. Side chain conformation for the Lys residue(s) of the best ranked NMR derived structures is resolved by using the rotamer library of Swiss Pdb Viewer [33]. This shows that the RMS deviation for the first four residues (‘C^α^NN’ segment: Leu1, Gly2, Lys3 and Gln4) are small (∼1.1 Å, with respect to C^α^-atom) when compared to the corresponding segment of the crystal structure (residue 107–110 of 1MUG) and named as ‘**experimental NMR structure**’ (Figure S1).

However, the 5-residue sequence (SCPS224Ac) of the ‘experimental NMR structure’ has been obtained by truncating the anchor helix part of the ‘experimental NMR structure’ of CPS224Ac.

#### b. Model structures

The ‘**native model’ structure** [the φ, ψ dihedral angles of the first four residues (‘C^α^NN’ segment) at the N-terminus of 18-residue chimeric peptides (CPS224Ac, CPS226 and CPS228) have been fixed to the φ, ψ dihedral angles of the individual residues found in the respective crystal structure : pdb (protein data bank) code 1MUG, 1YCC, 1JW9 respectively (Table 1) [1], [34], [35]] and the ‘**extended model’ structure** [the φ, ψ dihedral angles of the first four residues (C^α^NN segment) at the N-terminus of 18-residue chimeric peptides (CPS224Ac, CPS226, & CPS228) have been fixed to 180°, 180°] have been generated using the Accelrys Discovery Studio 2.5.5 [36] where in both the cases remaining 14 residues at the C-terminus have been fixed as right handed α-helices (φ = −57° & ψ = −47°). The backbone geometry of both the model structures has been validated using the DSSP program [37].

Short five residue sequences (SCPS224Ac, SCPS226 and SCPS228) of ‘native model’ structure and that of ‘extended model’ structure have been obtained similarly by truncating the anchor helix part from the respective conformation of CPS224Ac, CPS226 and CPS228.

### II. Molecular Docking Experiment

Molecular docking (rigid docking) experiments have been performed on the peptide sequences having different conformation at the ‘C^α^NN’ motif with both the sulfate and the phosphate ion.

#### a. Intrinsic affinity for anion(s)

**a.i. Peptide CPS224Ac.**

**Figure 1:**
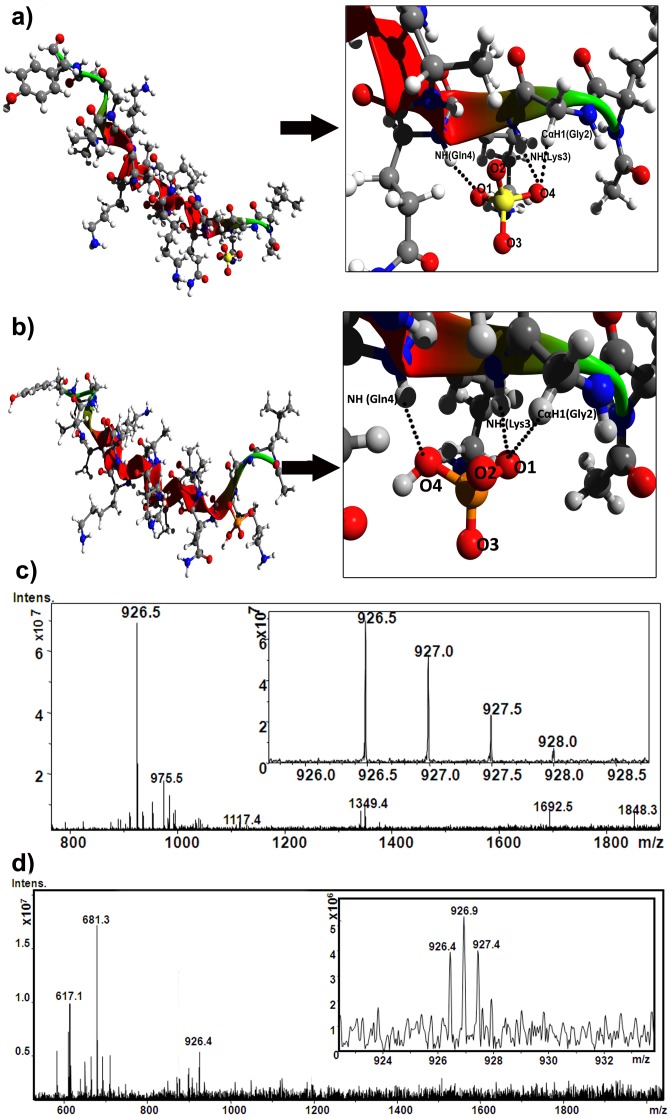
Representation of interaction of anion with ‘C^α^NN’ motif of CPS224Ac. a) Lowest energy conformer of the 250 sulfate ion docked structures of ‘experimental NMR’ structure along with its detailed representation of interactions showing that ‘C^α^NN’ motif segment acts as the only recognition site for sulfate ion; b) lowest energy conformer of the 250 phosphate ion docked structures of ‘experimental NMR’ structure along with its detailed representation of interactions showing that ‘C^α^NN’ motif segment acts as the only recognition site for phosphate ion; c) ESI-MS result showing the binding of sulfate ion with CPS224Ac (Inset: isotopic distribution of m/z 926.5 showing the difference of 0.5, indicating doubly charged species); d) ESI-MS result showing the binding of phosphate ion with CPS224Ac (Inset: isotopic distribution of m/z 926.4 showing the difference of 0.5, indicating doubly charged species).

**a.i.1. The ‘experimental NMR structure’.** Molecular docking experiments, using AutoDock 4.2 software [38], [39], have been performed between the ‘experimental NMR structure’ of CPS224Ac [25] and the anion(s) (separately with the sulfate and the phosphate ion) using several grid size to identify the site for recognition of anion in the peptide and to get an idea about the associated parameters of interaction. The results show that, in all the respective 250 iteration for individual sulfate and phosphate ion, for all the grid size only the ‘C^α^NN’ motif segment, present at the N-terminus of the context free helical peptide, can recognize the sulfate/phosphate ion through non-covalent interaction mediated through H-bond (hydrogen bond) (Figure 1a, b). MGL Tools (version 1.5.4) [40], used for monitoring the interaction of anion (sulfate/phosphate ion) with the ‘experimental NMR structure’ of peptide CPS224Ac, confirms that, for both the anions out of its four oxygen atoms, two are interacting simultaneously with the constituent atoms of the ‘C^α^NN’ motif (C^α^-H atom of Gly2, main-chain N-H atom of Lys3 and main-chain N-H atom of Gln4) of the related region of peptide CPS224Ac [Figure 1a, b], as observed in the respective crystal structure (C^α^-H atom Gly108, main-chain N-H atom of Lys109 and main-chain N-H atom of Gln110) of DNA Glycosylase (1MUG) (Figure S2). It is also found that out of these two interacting oxygen atoms, in each set of the respective 250 docked conformers, one oxygen atom is interacting concurrently with the C^α^-H atom of Gly2 and the main chain N-H atom of Lys3, while the other interacting oxygen atom has shared its contribution only to the main-chain N-H atom of Gln4, as observed in the crystal structure of 1MUG and proposed for the ‘C^α^NN’ motif [1], [13]. This result supports the observation of our earlier NMR study on CPS224Ac [25] which showed that upon interaction with the sulfate ion the chemical shift value of the main-chain N-H along with C^α^H atom of Lys3 and the main-chain N-H along with C^α^H atom of Gln4 were only altered (downfield shift observed for N-H, while upfield shift observed for C^α^H when compared to those individual in the absence of the sulfate ion), while others including ε-CH_2_ and side chain NH_2_ of Lys(s) remained unchanged (Figure S1). However, NMR results cannot locate the exact nature of the interaction of oxygen atoms of the sulfate ion, which could be acquired in details by this docking experiment.

**Figure 2:**
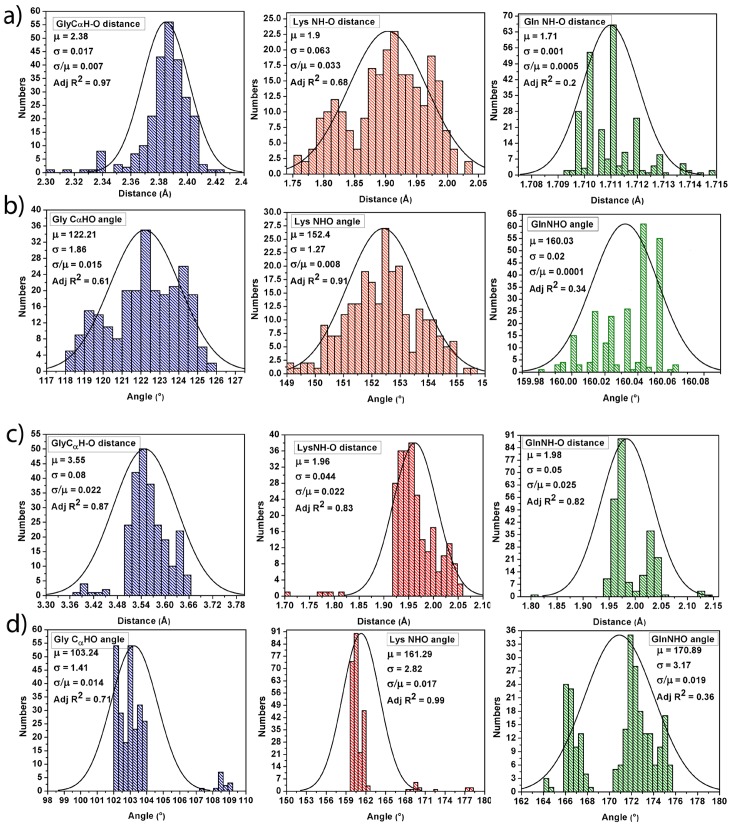
Distribution of H-bond distance and angle constraints obtained from interaction of anion(s) and ‘experimental NMR’ structure of CPS224Ac. (a) Distribution of (X)H—O distance for interaction with sulfate ion; (b) distribution of <X-H—O angle for interaction with sulfate ion; (c) distribution of (X)H—O distance for interaction with phosphate ion; (d) distribution of <X-H—O angle for interaction with phosphate ion (where X = C^α^_−1_/N_0_/N_+1_). The mean (μ) value, standard deviation (σ) value and goodness of the fit (adjusted R^2^) of each distribution are mentioned. The ratio of σ/μ is shown to emphasize that there is very little spread about the mean value.

**Interacting parameters.** Interaction between the ‘C^α^NN’ (C^α^_−1_N_0_N_+1_) motif of the ‘experimental NMR structure’ of CPS224Ac and the sulfate/phosphate ion is mediated through classical H-bonds where the C^α^_−1_-H—O H-bond pertains to be weak type [41], [42], while the two N-H—O, H-bonds are as of the conventional (moderate/strong) type, as observed in the respective crystal structure [13]. Distances and angles constraints between each oxygen atom of the sulfate (SO_4_^2−^)/phosphate (HPO_4_^2−^) ion and the constituent atoms of the ‘C^α^NN’ motif (C^α^-H atom of Gly2, main-chain N-H atom of Lys3 and main-chain N-H atom of Gln4) for each set of respective 250 docked conformers of the ‘experimental NMR structures’ of CPS224Ac are calculated (Figure 2a,b,c,d and Table 2) to describe the H-bond interactions, as in the crystal structures wherein the interactions are calculated on the basis of distance ((X)H—O<3 Å) and angles (X-H—O >90°) constraints [13].

**Table 2:** Interaction parameters between the sulfate/phosphate ion with the related ‘C^α^NN’ segment of the chimeric peptides in a context free system (250 docked structures of the individual conformation) are described in terms of X-H—O (where X = C^α^_−1_/N_0_/N_+1_) distances(**Å**) and angles(°) indicating the nature of H-bond formation (mean value of the parameters in parenthesis).

Peptides	Conformations at C^α^NN segment	Sulfate ion							Phosphate ion						
		(X)H…O Distance (Å)			<X-H…O Angle (°)			Binding free energy (Kcal/Mol)	(X)H…O Distance (Å)			<X-H…O Angle (°)			Binding free energy (Kcal/Mol)
		C_−1_^α^	N_0_	N_+1_	C^α^_−1_	N_0_	N_+1_		C_−1_^α^	N_0_	N_+1_	C^α^_−1_	N_0_	N_+1_	
CPS224Ac	NMR	2.30–2.42 (2.4)	1.75–2.04 (1.9)	1.70–1.71 (1.71)	118–125.67 (121.36)	149.23–155.5 (152.4)	159.95–160.06 (160.03)	−3.96	2.92–3.66 (3.54)	1.70–2.05 (1.96)	1.80–2.14 (1.98)	102.06–109.17 (103.24)	159.64–177.98 (161.29)	164.03–175.52 (170.89)	−3.48
	Native	2.86–3.15 (2.94)	2.29–2.61 (2.46)	1.73–1.80 (1.75)	104.75–108.38 (105.38)	100.01–102.00 (100.94)	169.66–177.63 (174.8)	−3.34	3.28–3.46 (3.36)	2.27–2.46 (2.33)	1.86–1.93 (1.92)	104.58–106.89 (105.52)	104.47–106.03 (105.51)	169.6–175.68 (171.3)	−2.40
	Extended	5.01–5.18 (5.16)	2.52–3.51 (3.46)	2.7–3.67 (3.51)	108.14–111.47 (111.13)	104.74–121 (108.48)	66.33–76.1 (67.84)	−1.67	3.27–3.71 (3.44)	2.1–2.2 (2.15)	4.32–4.71 (4.63)	110.59–112.48 (111.62)	141.89–146.02 (144.83)	33.82–43.85 (39.24)	−1.32
CPS226	Native	2.79–3.07 (2.86)	2.60–2.72 (2.65)	1.63–1.71 (1.66)	104.12–110.42 (107.07)	96.77–98.37 (97.25)	173.33–177.8 (176.00)	−3.37	2.43–3.76 (2.88)	1.77–2.17 (1.88)	1.94–2.12 (2.00)	127.65–142.5 (138.24)	151.63–165.24 (161.98)	166.40–170.20 (167.79)	−2.53
	Extended	2.72–3.17 (3.08)	1.96–2.22 (2.12)	5.7–5.88 (5.81)	117.27–117.8 (117.71)	146.27–149.54 (149.38)	36.04–39.84 (38.09)	−1.98	3.54–4.1 (3.78)	1.85–2.3 (2.11)	5.46–5.76 (5.71)	109.55–115.23 (113.18)	141.12–150.46 (145.06)	39.01–42.04 (40.03)	−1.53
CPS228	Native	3.63–3.92 (3.77)	1.88–2.16 (1.99)	1.78–2.06 (1.83)	119.89–124.36 (122.15)	135.88–144.5 (141.14)	169.9–179.23 (176.12)	−2.46	3.59–4.07 (3.87)	1.76–2.19 (1.98)	1.69–2.18 (1.76)	112.39–116.7 (115.13)	134.95–161.5 (139.3)	164.16–173.82 (165.75)	−1.60
	Extended	5.92–6.19 (6.06)	4.57–4.72 (4.64)	1.77–1.88 (1.81)	85.62–88.41 (87.15)	50.68–55.21 (53.54)	150.54–154.95 (153.33)	−1.93	3.46–3.77 (3.65)	1.9–2.13 (2.07)	5.35–5.57 (5.45)	95.43–104.95 (100.09)	165–175.35 (172.54)	42.33–49.57 (46.23)	−1.48

The ranges of the parameters comply well with those obtained for respective crystal structures and described by Denessiouk et al., in 2005. The estimated binding free energy gives a measure of relative stability of interaction (relative affinity for the anion) which largely depends on the conformational status of the ‘C^α^NN’ segment.

The (X)H—O (where X = C^α^_−1_/N_0_/N_+1_) distances (<3 Å) and the almost linear nature of the <X-H—O (where X = C^α^_−1_/N_0_/N_+1_) angles (>120°) (Table 2) obtained for the individual 250 anion (sulfate/phoshate) docked conformers of CPS224Ac (‘experimental NMR structure’) have been fitted with a ‘normal distribution’ (Figure 2 a, b, c, d) for which the mean value (μ) along with the standard deviation (σ) have been shown in the inset. The goodness of fit of the curves, as given by values of the adjusted R^2^ in figure 2 (a, b, c, d), show that for both the anions, the distribution of (X)H—O distances and <X-H—O angles (where X = C^α^_−1_/N_0_/N_+1_) largely follow the ‘normal distribution’, where almost the entire range of values cluster around the mean value with very little variance and comply well with the distribution ranges observed in such interactions in the crystal structures reported in the literature [13]. The observed low value of σ/μ (∼10^−2^) highlights that the mean value can be approximated as the actual value which matches well with those observed in the crystal structure of DNA Glycosylase (pdb ID 1MUG) [1] resulting from the interaction of a sulfate ion (Table S1, Figure S2). The interaction, thus mediated through H-bond, indicates that the formation of structures are well justified and therefore be a good candidate for MD simulation.

Similar ranges of interacting parameters [(X)H—O distances and <X-H—O angles (where X = C^α^_−1_/N_0_/N_+1_)] are also observed for each set of 250 individual docked conformations of SCPS224Ac (the 5-residue short ‘experimental NMR structure’) for both the anions. This validates the idea that ‘C^α^NN’ sequence with comparable conformation, even while the sequence is very short, can recognize the anion through H-bond interaction in a similar manner (Table S2 and Figure S3).

**a.i.2. The ‘model structures’.** Identical site (C^α^-H atom of Gly2, main-chain N-H atom of Lys3 and main-chain N-H atom of Gln4 of ‘C^α^NN’ motif segment present at the N-terminus) for recognition of both the sulfate and the phosphate ion through non-covalent (H-bond) manner, is observed for both the ‘native model’ (Figure S4, S5) and the ‘extended model’ structure of CPS224Ac in molecular docking [AutoDock software version 4.2 [38], [39]] experiments. However, the resulting values of the interacting parameters vary with the conformation at the ‘C^α^NN’ segment (rigid docking) as well as the interacting anion, measured from X-H—O (where X = C^α^_−1_ Gly2/N_0_ Lys3/N_+1_ Gln4) angles and distances constraints (Table 2). MGL Tools (version 1.5.4) [40] indicates that out of the four oxygen atoms of the sulfate/phosphate ion, two are interacting simultaneously with the constituent atoms of the ‘C^α^NN’ motif (C^α^_−1_ Gly2/N_0_ Lys3/N_+1_ Gln4) only. Out of these two oxygen atoms, one is interacting concurrently with the C^α^-H atom of Gly2 and the main-chain N-H atom of Lys3, while the other interacting oxygen atom shares its contribution only to the main-chain N-H atom of Gln4; similar to that observed in the ‘experimental NMR structure’, reported crystal structure (1MUG) [1] and proposed for the ‘C^α^NN’ motif [13].

For the short model structures (native as well as extended) similar interaction among the sulfate/phosphate ion and the ‘C^α^NN’ motif segment is observed. However, the corresponding values of the parameters vary according to the conformation of ‘C^α^NN’ motif segment and the interacting anion, as observed for its respective 18-residue chimeric analogue (Table S2).

**Interacting parameters.** Similar ranges of (X)H—O distance and <X-H—O angle constraints (where X = C^α^_−1_/N_0_/N_+1_) are obtained for the ‘native model structure’ of CPS224Ac (Table 2) as well as for its ‘short native model structure’ (SCPS224Ac) (Table S2) for each set of respective 250 docked conformers when docked separately with sulfate and phosphate ion, pertaining weak C^α^_−1_-H—O and conventional N-H—O, H-bond interaction. However, comparatively poor interaction with the anion(s) is observed for the ‘extended model structures’ of CPS224Ac as well as its ‘short extended model structures’ (SCPS224Ac) although the site for recognition of anion(s) is identical (C^α^-H atom of Gly2, main-chain N-H atom of Lys3 and main-chain N-H atom of Gln4). Comparatively large (X)H—O distance constraints and quite low values of <X-H—O angle (deviated more from linearity) (where X = C^α^_−1_/N_0_/N_+1_)] (Table 2 and Table S2), with respect to those of the ‘experimental NMR structure’ and the ‘native model structure’, support the conclusion. This corroborates the idea that ‘helical’ conformation at the ‘C^α^NN’ motif segment triggers favourable interaction with the anion.

**a.i.3. Estimated free energy of binding/Calculated apparent binding energy.** Magnitude of the ‘binding free energy’ although varies depending on the conformational status of the ‘C^α^NN motif’ segment as well as the interacting anion (Table 2), single cluster of ‘free energy of binding’ (AutoDock module calculated this using the inbuilt program within the software) [43] is obtained for each individual set of respective 250 docked conformers obtained from the ‘experimental NMR structures’, ‘native model structures’ and ‘extended model structures’ of CPS224Ac as well as their short sequence (SCPS224Ac) for both the sulfate and the phosphate ion. This clearly establishes the presence of only one site (‘-Gly-Lys-Gln-’ segment of the ‘C^α^NN’ motif at the N-terminus of CPS224Ac and SCPS224Ac) for anion recognition in the overall sequence and thus emphasizes the ‘local’ nature of interaction.

Average value(s) of ‘estimated free energy of binding’ obtained from the interaction of sulfate/phosphate ion and the different structures of CPS224Ac reported in Table 2 (for SCPS224Ac, Table S2) may not be an absolute realistic measure; however, it reflects the relative estimate of anion binding potential of the ‘C^α^NN’ motif at its different conformational state as well as its preference of anion. The observed difference of ∼2 Kcal/mole in average free energy of binding between the ‘experimental NMR’/‘native model’ conformer and that of the ‘extended model’ structure accentuate that ‘helical’ conformation at the ‘C^α^NN’ segment has higher affinity for anion recognition in comparison to ‘non-helical’ conformation (difference in the backbone dihedral angle constraints of the interacting segment). This would validate the rationality of the extension of helical structure at the ‘C^α^NN’ segment from non-helical one, to accommodate the tetrahedral sulfate/phosphate ion for favourable interaction, as proposed by Denessiouk et al [13].

**a.ii. Peptide CPS226 and CPS228.**

**The ‘model structures’.** Appearance of single ‘binding free energy’ cluster obtained for each set of respective 250 docked conformers for both the ‘model structures’ (‘native’ and ‘extended’) of CPS226 and CPS228 peptides along with their short sequences confirms that ‘C^α^NN’ segment (Ser-Ala-Lys for CPS226/SCPS226 and Gly-Gly-Leu for CPS228/SCPS228) [34], [35] appended at the N-terminus of the sequences act as the only interacting site for the sulfate/phosphate ion as observed for CPS224Ac (and also SCPS224Ac) (Figure S3, S4, S5). Magnitudes of the average ‘binding free energy’ as well as X-H—O (where X = C^α^_−1_/N_0_/N_+1_) interacting parameters (distance and angles) vary depending on the conformation as well as the sequence of the ‘C^α^NN’ motif segment in the peptide and the interacting anion (Table 2) as observed for CPS224Ac.

Similar to our observation in CPS224Ac and that reported in the respective crystal structures of 1YCC and 1JW9 (Figure S2) [34], [35], out of the four oxygen atoms of the sulfate/phosphate ion, two are interacting simultaneously with the constituent atoms of ‘C^α^NN’ motif of the related region of peptide CPS226 & SCPS226 (C^α^-H atom of Ser2, main chain N-H atom of Ala3 and main chain N-H atom of Lys4) and that of peptide CPS228 & SCPS228 (C^α^-H atom of Gly2, main chain N-H atom of Gly3 and main chain N-H atom of Leu4) (Figure S3, S4, S5). The distribution of (X)H—O distances and <X-H—O angle (where X = C_−1_^α^/N_0_/N_+1_) for each set of 250 anion (sulfate/phosphate) docked conformers of the individual peptides in their ‘native model’ structures, by and large, follow normal distributions having almost the entire range of values cluster around the average value (data not shown) and comply well with the parameters resulting from the interaction of a sulfate ion in the respective crystal structure (1YCC and 1JW9 respectively) [34], [35] (Table 2, Table S1). Short sequences of both the peptides also show similar interactions (Table S2, Figure S3).

However, comparatively poor interaction of anion (sulfate/phosphate ion) with the ‘C^α^NN’ motif segment in the ‘extended model’ structures is observed for both the sequences (CPS226, SCPS226 and CPS228, SCPS228) from their lower binding energy and the weak (X)H—O interaction parameters [larger values of X-H—O distance and lower values of <X-H—O angle constraints (where X = C_−1_^α^/N_0_/N_+1_)] (Table 2 and Table S2) as observed for the CPS224Ac and SCPS224Sc.

#### b. Interactions at a glance

In spite of the presence of three Lys residues in the anchoring helix and regardless of the hydrophilic or hydrophobic nature of amino acids in the ‘C^α^NN’ motif, it is found that in all the peptides only the consecutive C^α^_−1_, N_0_ and N_+1_ of the main chain atoms of the ‘C^α^NN’ motif located at the N-terminus of the anchor helix participate in anion recognition. This would be considered that the positive end of the helix macro-dipole may contribute to the anion recognition [44]. However, a more plausible alternative explanation for anion recognition can be interaction of anion with the localized micro-dipoles arising out of the NH (CONH) and C^α^H of the protein/peptide main-chain through H-bond which is to a large extent electrostatic in nature [45], [46]; as changes in charge at the C-terminus of helix have been found not to affect the sulfate binding in proteins [47]. In addition, the greater acidic nature of H of main chain CONH in comparison to that of the side chain ε-NH_2_ of Lys, would allow the main chain NH (as CONH) for higher anion recognition over the side chain NH_2_, through formation of stronger H-bond (N-H—O), which is explicitly observed in our study. This observation is in contrast with the recent anion binding study of Lys incorporated hexapeptides, where Bianchi et al [16] have ‘unexpectedly’ observed the participation of Lys side-chain ε-NH_2_ along with main chain CONH in the interaction with HPO_4_^2−^ anion.

Thus the resulting X-H—O (X = C^α^_−1_, N_0_ and N_+1_) distances and angle parameters appear on the recognition of both the sulfate and the phosphate ion separately by the ‘C^α^NN’ segment through H-bond, (Table 2, Figure 2 and Table S2), which comply well with those parameters reported for the respective crystal structures [13] (Table S1), emphasizes that the ‘C^α^NN’ motif segment has an intrinsic affinity for the anions and the information regarding anion binding is embedded in its local sequences. Thus one can justify the ‘conserved nature’ of the ‘C^α^NN’ sequence for anion recognition through ‘local’ interaction.

However, a consensus can be drawn from the comparison of the binding free energy (gives a relative estimate for the feasibility of interaction) obtained from the docking experiments (Table 2, Table S2). Interaction with the sulfate ion is likely to be preferred by the ‘C^α^NN’ motif segment, in comparison to that of the phosphate ion although both the interactions are thermodynamically favorable. This is similar to the observation of Demuth et al [48] which showed the dominance of interaction of the sulfate ion over the phosphate ion with a designed oligopeptide where hydrophilic side chains (Ser, Arg) through salt bridges play crucial role in intermolecular interaction. Moreover, the observed preference for sulfate ion interaction also supports the fact that although ‘C^α^NN’ motif is considered as novel structural motif in protein for recognition of phosphate ion, yet during crystallization of these proteins in several cases sulfate ion occupies the positions of interaction as anions [13].

Our study thus highlights the significance of the conserved nature and the conformational preference of the ‘C^α^NN’ motif sequence for recognition of the anion(s) as suggested by Denessiouk et al. [13]. Comparatively ‘poor’ interaction between the ‘extended model’ structure of all the peptides (CPS224Ac and SCPS224Ac; CPS226 and SCPS226; CPS228 and SCPS228) and the anion(s) (Table 2 and Table S2), clearly corroborates that for thermodynamically favorable anion recognition through H-bond by the ‘C^α^NN’ motif, helical conformation at this segment is required. This would rather strongly justify the extension of ‘helical’ structure at the ‘C^α^NN’ motif segment from the ‘non-helical’ structure as a result of co-operative effect initiated by the approach of anion towards the motif segment and augmented by the attached helix as observed in the interaction of sulfate ion with CPS22Ac using CD and NMR spectroscopy [25]. This point is further clarified below from MD simulations.

### III. Molecular Dynamics Experiment

Computer simulated Molecular Dynamics (MD) study is one of the best theoretical approaches for investigating the protein/peptide conformational microstates as well as the atomic-level details during the binding interaction from kinetic approach [49]–[51]. Moreover, simulations with explicit water help to obtain more realistic picture of the interaction.

**Figure 3:**
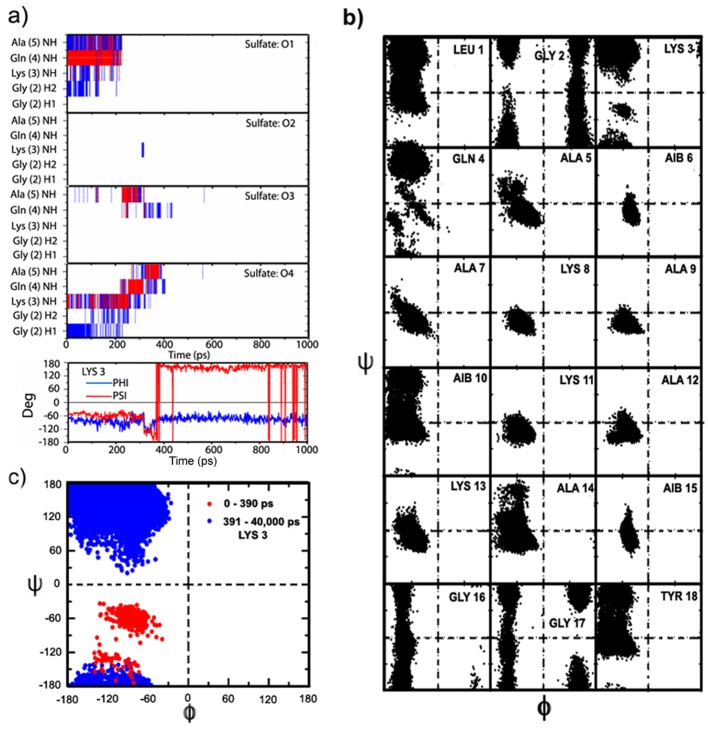
MD simulations (for 40 ns at 276K) of the sulfate ion-bound conformer of the ‘experimental NMR structure’ of CPS224Ac. a) Partial representation (first 1 ns of total 40 ns simulation) of sulfate ion interaction with the C^α^NN′ motif segment of CPS224Ac (blue lines indicate weak interaction while red line indicate strong H-bond) along with the detail monitoring of backbone dihedral angles (φ, ψ) of Lys3 with respect to time in the MD trajectory; b) Ramachandran plot of the backbone dihedral angle distributions (φ, ψ) of all the residues during the MD simulation (40 ns) showing perturbation takes place only in the C^α^NN′ motif segment; c) Distribution of backbone dihedral angles (φ, ψ) of Lys3 residue as Ramachandran plot during the 40 ns MD-simulation, emphasizing its existence in helical conformation only during interaction of sulfate ion with C^α^NN′ motif segment peptide.

**Figure 4:**
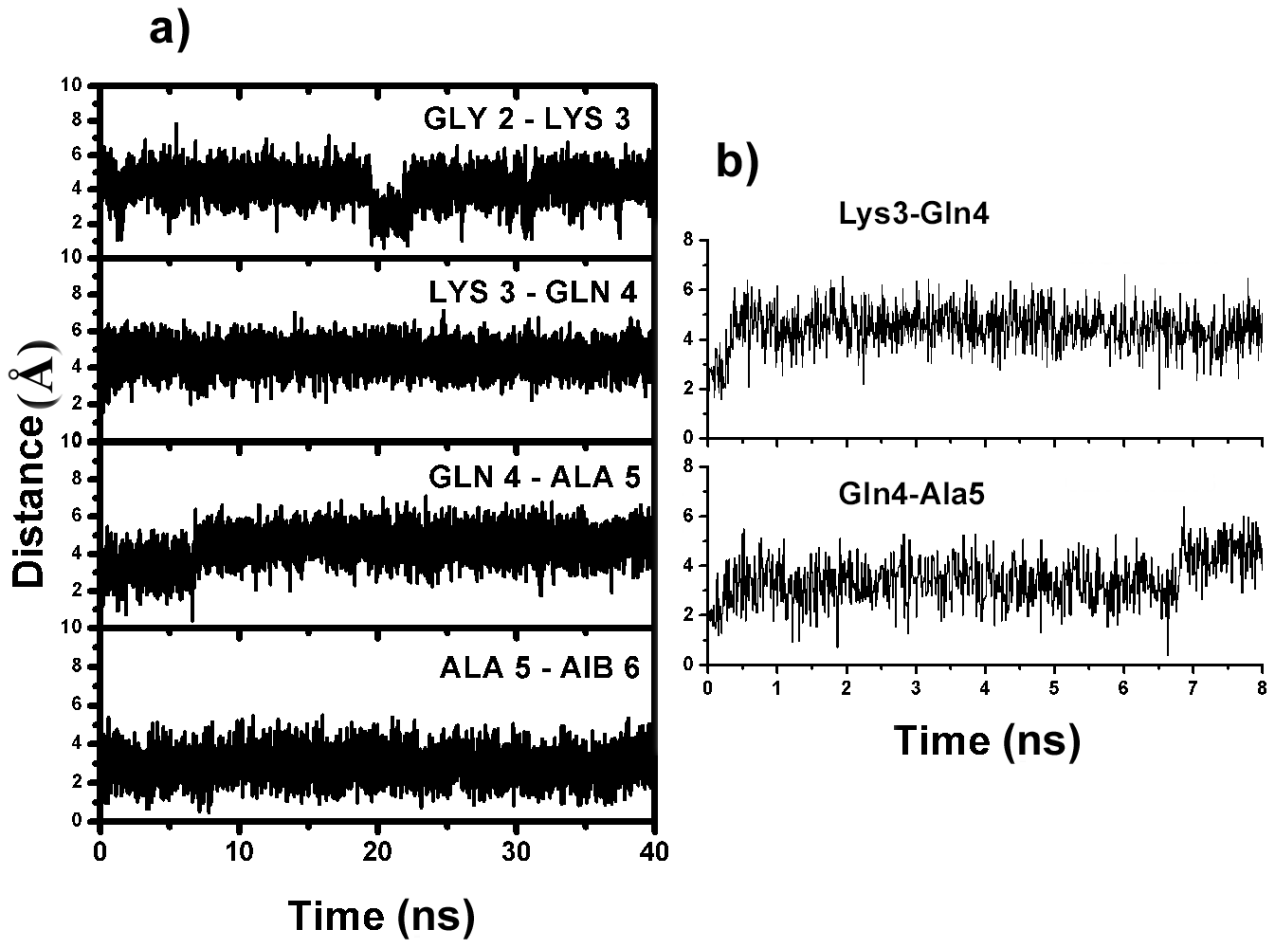
Details of NH—NH_(i,i+1)_ distance(s) around the ‘C^α^NN’ region during the MD simulation. a) For 40 ns and b) first 8 ns; showing the destabilization of helical conformation at the ‘C^α^NN’ segment in the MD trajectory.

To investigate anion induced helical conformation at the ‘C^α^NN’ motif segment, MD simulations (for 40 ns at 276K) are carried out starting from the sulfate ion-bound conformer(s) of the ‘experimental NMR structure’ of CPS224Ac obtained from the docking experiments. Results, summarized in Figure 3 and Figure 4, show that during ‘sulfate-peptide’ interaction (∼400 ps), out of the two interacting oxygen atoms of the sulfate ion, one oxygen atom is interacting concurrently with the C^α^-atom of Gly2 and the main-chain N-atom of Lys3, while the second interacts only to the main chain N-atom of Gln4 of the ‘C^α^NN’ motif through H-bond (Figure 3a), similar to that found in 1MUG (Figure S2) and reported for anion-‘C^α^NN’ motif interaction [13] (a few snapshots of the MD trajectory is represented in Figure S6). This establishes that the ‘C^α^NN’ anion-binding motif comprised of C^α^_−1_, N_0_ and N_+1_ backbone atoms from three consecutive residues (Gly-Lys-Gln), binds the anion through ‘local’ interactions even in a context free system, as observed in the docking experiments as well as NMR experiment [25] of CPS224Ac with sulfate ion. However, once the sulfate ion leaves the ‘C^α^NN’ motif, it remains as free sulfate ion till 40 ns and does not bind to any group/atom, even to the ε-NH_2_ group of Lys; which is consistent with the single interacting site, observed in the docking experiment. To confirm the statistical significance of such ‘sulfate ion-‘C^α^NN’ interaction’, additional MD experiments have been pursued with bound anion for different time steps (20–40 ns) with different initial velocities. Similar type of interactions of sulfate ion with the ‘native model’ structure of CPS224Ac (residence time ∼1000 ps) at 276K (Figure S7) is also observed.

To assess the residue specific conformational microstates observed during the simulation (∼40 ns) of ‘experimental NMR structure’ of CPS224Ac with the sulfate ion, the backbone dihedral angles (φ, ψ) are plotted on Ramachandran plot (Figure 3b). It can be seen that the backbone dihedral angles for the residues Ala5-Aib15 are mostly confined to the right-handed helical conformation throughout the simulation. However, an appealing scenario is observed for the ‘C^α^NN’ residues especially for Gln4 and Lys3, although the starting conformation (sulfate ion docked structure) of -Gly-Lys-Gln- is β-α_R_-α_R_ as described by Denessiouk et al. [13]. The backbone dihedral angles (φ, ψ) of Gln4 (Figure 3b, details not shown) is distributed among the right-handed helical conformation (up to ∼400 ps), non-canonical helical conformation (400∼7000 ps) along with the PPII conformation (∼7000–40,000 ps), while for Lys3, the φ and ψ values remain in the right-handed helical conformation [φ = −70° (±15), ψ = −57° (±12)] till the sulfate ion interacts to the ‘C_α_NN’ segment (Gly-Lys-Gln) of CPS224Ac (∼400 ps). Once the sulfate ion ceases to interact with the peptide segment, the φ and ψ values of Lys3 instantaneously undergo an immediate transition from the helical form to the PPII conformation [φ = −70° (±15), ψ = 150° (±20)](Figure 3a, c) and remains there till the end of simulation (400–40,000 ps). Similar distributions of backbone dihedral angles are obtained for the ‘native model’ structure with sulfate ion interaction during the MD simulations (data not shown).

NH—NH (i,i+1) distance parameter (<4 Å) is a good measure for prevalence of the helical conformation [31]. Tracking the interaction using Euclidean distance measure, it is observed that the NH—NH distance between Lys3-Gln4 remains <2.5 Å till the sulfate ion is attached to the motif sequence (∼400 ps). This, suddenly increases to >4.5 Å once the sulfate ion moves apart (∼400 ps) from the peptide segment and remains distant (>4.5 Å) till the end of the simulation (Figure 4). An increase in the NH—NH distance between Gln4-Ala5 from ∼2.5 Å to ∼3.8 Å is also observed once the interaction of the sulfate ion fades away. The concurrent increase in the NH—NH distance between Lys3-Gln4 and Gln4-Ala5 reflects the destabilization of the helical population at ‘C^α^NN’ motif resulting from the loss of the sulfate ion interaction. However, the restriction of Gln4 (φ, ψ) conformation into non-canonical helical form even in absence of the sulfate ion (up to ∼7 ns), supported by the NH—NH distance between Gln4-Ala5 (Figure 4), may be attributed to the presence of the neighboring helicogenic α-amino isobutyric acid (Aib/B) residue (Aib6) [29], [30]. This effect of Aib on Gln4 can be inferred from the MD simulations of the short five-residue sequence SCPS224Ac with the sulfate ion, where ejection of the sulfate ion from the ‘C^α^NN’ segment (∼400 ps) immediately transits Gln4 towards a non-helical conformation (PII) (Figure S8).

The MD results thus corroborate that the helical conformation of the ‘C^α^NN’ motif segment embedded in the context free peptide sequence is sustained only during the time of sulfate ion interaction [for ‘experimental NMR structure’ ∼400 ps, while for ‘native structure’ ∼1000 ps; Figure 3 and Figure S7]. On ejection of the sulfate ion, the helical conformation of the ‘C^α^NN’ motif undergoes an immediate transition to a non-helical ‘extended structure’ and at no point the segment has acquired any nascent form of helical structure despite a long simulation time (40 ns). This strongly suggests that the ‘C^α^NN’ motif peptide segment itself has limited or no intrinsic helical propensity and validates that there is a cooperative transition involving the C^α^NN motif into helix, induced by the anion binding. In this new conformation the length of the anchoring helix gets extended by one additional turn towards the N-terminus as proposed for ‘C^α^NN’ motif by Denessiouk et al. [13], and observed in CPS224Ac-sulfate ion interaction through NMR spectroscopy [25]. The observed m/z values [for the sulfate ion (m/z 926.5 with isotopic distribution difference of 0.5 indicating doubly charged species) and for the phosphate ion (m/z 926.4 with isotopic distribution difference of 0.5 indicating doubly charged species)] obtained in ESI-MS experiments confirm the relevant interaction of sulfate and phosphate ion with the peptide CPS224Ac (Figure 1c, d). As biophysical experiments ensure the anion recognition, the loss of the sulfate ion in MD experiments may be interpreted as an indicator for lack of intrinsic helical propensity of the ‘C^α^NN’ segment rather than lack of anion binding; because it is obvious that the force-field is not successful in encapsulating the experimental observation.

### IV. Conclusion

The current work primarily substantiates that information regarding the anion recognition by the ‘C^α^NN’ motif (found in naturally occurring proteins) is embedded within its primary structure and justify the ‘conserved nature’ of the sequences in ‘C_α_NN’ segment. Complementary computational techniques along with ESI-MS experiments clearly establish that even in the absence of a proteinaceous environment (‘C^α^NN’ segments appended as a part of an isolated helix, without any tertiary structural effect) the ‘C^α^NN’ motif sequences can recognize the sulfate/phosphate ion through ‘local’ interactions corroborating its intrinsic affinity for the anions. In the MD simulation, the detailed view of loss of the helical structure at the N-terminus immediately after fading out of interaction of the anion (sulfate ion), confirms an accompanied conformational switching from the non-helical to the helical state at this ‘C^α^NN’ anion-binding motif segment upon interaction with the anion, as proposed in the literature. The binding free energy (negative value) and the interaction parameters obtained from the computational study suggest that the anion recognition/binding is thermodynamically favorable; however, it depends on the conformation of the motif segment and the nature of the interacting anion. The helical conformation induced at this four-residue segment of the ‘C^α^NN’ anion-binding motif upon anion binding may occur as a result of the co-operative effect initiated during the approach of anion towards the ‘motif’ segment and be augmented by the anchoring helix which may have an implication for nucleation of folding. Moreover, using these computational techniques the detailed overview obtained about the stereochemistry of the anion recognition, which is really difficult to acquire from the ^1^H-NMR data as the results obtained in NMR experiments is a weighted average, are in consistent with what is proposed about the ‘C^α^NN’ anion-binding structural motif in proteins and validate our previous results of the sulfate ion interaction with the peptide CPS224Ac as monitored by NMR and other spectroscopic techniques [25]. This study will help our understanding of the influence of sequence/structural context of anion binding in proteins which not only shed light on the aspects of protein folding but also provide guidelines for designing ‘peptide-based model scaffold’ host-receptors for anion-binding sites. This would likely to be adaptable for novel recognition purpose at the molecular level and to be used in various biotechnological applications.

## Methods

### Docking

To characterize molecular recognition and binding of sulfate (SO_4_^2−^) and phosphate (HPO_4_^2−^) ion by the ‘C^α^NN’ anion recognition segment with different conformations in a series of designed peptide sequences, AutoDock 4.2 software package [38], [39] is used using Mac OS X 10.6.6 as an operating system. AutoDock Tools (version 1.5.4) is used to prepare the input files for docking experiments (rigid docking). Atomic coordinates of sulfate (SO_4_^2−^) and phosphate (HPO_4_^2−^) ion are obtained from protein data bank and docked separately with ‘experimental NMR structure’ and ‘native model structure’ along with the ‘extended model structure’ of 18-residue chimeric and short 5-residue sequences. All the hydrogen atoms are added and partial charges (Gasteiger charge) are assigned in the standard PDBQT file format. Different grid values [i.) first eight residues (up to Lys8) from the N-terminus acetyl along with their side chains and ii.) first five residues (up to Ala5) from the N-terminus acetyl with their side chains for all 18-residue sequences, while for all short sequence up to Ala5 including side chains from the N-terminus acetyl] are used to confirm the anion recognition site in the peptides. Although for both the grid values, identical site of interaction along with similar binding energy [e.g. for ‘experimental NMR structure’ of CPS224Ac −4.04 Kcal/mol for the grid value up to Lys8 (Grid points: 44(x) 44(y) 44(z)); while −3.96 Kcal/mol for grid value up to Ala5 (Grid points: 40(x) 40(y) 40(z))] are obtained in all the respective cases; however, for comparison of interaction parameters among the peptides (Table 2 and Table S2) results for the grid value containing up to Ala5 is shown (as there is only 5 residues in short sequence). A user-defined protocol of 150 randomly generated individuals with a maximum number of 2.5×10^7^ energy evaluations and a maximum number of 2.7×10^4^ generations is employed for running the docking programme. For each case 250 anion bound conformations (iteration) are generated using Genetic Algorithm (GA-LS) searches with a mutation rate of 0.02 and a crossover rate of 0.8. Validation of the docking results is satisfied using the same package of AutoDock.

All the images are developed using Avogadro (version 1.0.3) and Chimera (version 1.6.2) [52]. Further, the images are rendered using POV-Ray (version 3.6).

### MD Simulations

Molecular Dynamics studies are carried out on peptide-sulfate complexes – a. sulfate bound to CPS224Ac (‘experimental NMR’ structure and ‘native model’ structure); b. sulfate bound to SCPS224Ac (‘experimental NMR’ structure), using version 4.0 of the GROMACS package [53], [54] with the OPLSAA force field [55]. The starting structures of the complexes used in this molecular dynamics study are the lowest energy structure(s) of the sulfate (SO_4_^2−^) ion bound particular conformation of the peptide, obtained from docking experiments. The sulfate (SO_4_^2−^) ion is built using GaussView 5.0.8 [56] and the geometry optimization with partial charge calculation are done using gaussian09 [57] with the 6311G++(d,p) basis set.

The peptide-sulfate complex(s) is placed within a cubic box with a distance of approximately 1.0 nm between the periphery of the complexes and the sides of the box. The box is filled with pre-equilibrated SPC (simple point charge) water [58]. The charge neutrality of the system is achieved by the insertion of four Cl^−^ and two Na^+^ ions replacing six water molecules for the long sequence and one Cl^−^ and two Na^+^ ions replacing three water molecules for the short sequence respectively. Both the systems are subject to an initial energy minimization applying the steepest descent algorithm. The systems are then subjected to NVT (constant number of particles, volume and temperature) ensemble dynamics to allow the relaxation of the water molecules in the systems while the peptide-sulfate complexes is restrained to their initial coordinates with a force constant of 100 KJmol^−1^, followed by NPT (constant number of particles, pressure, temperature) equilibration [59] for 100 ps respectively. The systems are prepared by heating in increments of 50K for 20 ps until the desired temperature of 276K (3°C) is reached. Subsequently production MD simulations at constant pressure and temperature are carried out at 276K (3°C) with a time step of 2 fs using Berendsen coupling method [59]. The temperature and pressure time coupling constants used are 0.1 and 0.5 ps respectively. LINCS [60] algorithm is applied to constrain all bonds during the simulations. PME [61] method with a cut-off of 1.0 nm is used to calculate the non-bonded interactions and the neighbor-list is updated every 10 steps. The structures after every 1 ps are saved to the trajectory and are analyzed using various GROMACS utilities and custom written Perl and Fortran programs. The hydrogen bonding interaction between the sulfate ion and the peptides are calculated using a simple Coulomb point charge model adapted from the DSSP program [37] for calculating secondary structure from hydrogen bonds. For S = O—H-N interaction, the equation used is [E = 332(qSqH/rSH+qSqN/rSN+qOqH/rOH+qOqN/rON) Kcal/mol; where ‘r’ represents inter-atomic distances in Å and ‘q’ represents partial charge at each atom: qS = 1.6; qO = −0.9; qH = 0.3; qN = −0.5], and for S = O—H-C^α^ the equation used is [E = 332(qSqH/rSH+qSqC^α^/rSC^α^+qOqH/rOH+qOqC^α^/rOC^α^) kcal/mol, where ‘r’ represents inter-atomic distances in Å and ‘q’ represents partial charge at each atom: qS = 1.6; qO = −0.9; qH = 0.06; qC^α^ = −0.1]. E −0.5 to −1.0 kcal/mol and E≤−1.0 are considered as a weak [41], [42] and strong hydrogen bond respectively. The H-bond interaction is further validated from (X)H—O (X = C^α^_−1_, N_0_ and N_+1_) distance [(C^α^)H—O≤3 Å and (N)H—O≤2.7 Å] and angle [X-H—O≥90°] parameters. Structures are visualized with the program Chimera [52].

### ESI-MS

Binding of sulfate/phosphate added species of CPS224Ac (through m/z value) is studied in negative mode in ESI-FTMS instrument (Apex Ultra 70, Bruker Daltonics direct infusion mode) using H_2_O/CH_3_CN (1∶1) with 0.1% NH_3_.

## Supporting Information

Figure S1 **Effect of addition of sulfate ion to peptide CPS224Ac using NMR spectroscopy.** **a**) 1D spectra of (HN region) indicate that only K3 and Q4 suffer a change in chemical shift values (downfield shift). **b**) ΔδC^α^H (Hα secondary chemical shift in ppm) in absence as well as in presence of sulfate ion (identified through individual TOCSY experiment). **c**) Cluster of 10 best ranked NMR-derived structures of sulfate added CPS224Ac, generated from NMR constraints using programme DYANA
(TIF)

Figure S2 **Representation of interaction of sulfate ion with the ‘C^α^NN’ segment of the respective proteins found in the crystal structure deposited in PDB.**
(TIF)

Figure S3 **Representation of sulfate ion interactions with the ‘experimental NMR structure’ of SCPS224Ac and the ‘native model structure’ of SCPS228 showing that the ‘C^α^NN’ motif segment can recognize sulfate ion even in short sequences.**
(TIF)

Figure S4 **Representation of sulfate ion interactions with the ‘native model’ structure of CPS224Ac, CPS226 and CPS228 showing that the ‘C^α^NN’ motif segment at the N-terminus act as the only recognition site for sulfate ion in each case.**
(TIF)

Figure S5 **Representation of phosphate ion interactions with the ‘native model’ structure of CPS224Ac, CPS226 and CPS228 showing that the ‘C^α^NN’ motif segment at the N-terminus act as the only recognition site for phosphate ion in each case.**
(TIF)

Figure S6 **A few snapshots of MD trajectories (40 ns) showing the interaction of sulfate ion with the ‘C^α^NN’ motif in ‘Experimental NMR’ structure of CPS224Ac.**
(TIF)

Figure S7 **Partial representation of sulfate ion interaction with the C^α^NN′ motif segment of CPS224Ac (‘Native model’ structure) in Molecular Dynamics experiment at 276K (blue lines indicate weak interaction while red lines indicate strong H-bond), showing out of four oxygen two are simultaneously interacting with constituent main-chain atoms through H-bond.**
(TIF)

Figure S8 **Distribution of backbone dihedral angles (φ, ψ) of the Gln4 residue during the MD simulation of sulfate ion interactions with the ‘experimental NMR structure’ of SCPS224Ac emphasizing its existence in helical conformation during interaction of sulfate ion with ‘C^α^NN’ motif segment peptide and the role of Aib6 residue pertaining the non-canonical helical conformation of Gln4 in CPS224Ac in the absence of sulfate ion.** Two snapshots of the interaction are shown when the sulfate ion is close to ‘C^α^NN’ segment and apart from the segment.
(TIF)

Table S1 **Representation of interaction parameters ((X)H…O Distance (Å) and <X-H…O Angle (°)) of anion (sulfate ion) with ‘C^α^NN’ segment obtained from the respective crystal structures of proteins reported in PDB (^a^Barrett etal.,1998; ^b^Louie and Brayer 1990; ^c^Lake etal., 2001).**
(DOC)

Table S2 **Interaction parameters between the sulfate/phosphate ion with the related ‘C^α^NN’ segment of the short 5-residue peptides (truncated version of the 18-residue sequences) in a context free system (250 docked structures of the individual conformation) are described in terms of X-H—O (where X = C^α^_−1_/N_0_/N_+1_) distances (Å) and angles (°) indicating the nature of H-bond formation (mean value of the parameters in parenthesis).** The estimated binding free energy gives a relative affinity for anion which depends on the conformational status of the ‘C^α^NN’ segment.
(DOC)

## References

[1] Barrett TE, Savva R, Panayotou G, Barlow T, Brown T, et al (1998) Crystal structure of a G:T/U mismatch-specific DNA glycosylase:mismatch recognition by complementary-strand interactions. Cell (Cambridge Mass) 92: 117–129. 10.1016/s0092-8674(00)80904-6 9489705

[2] Cobessi D, Tete-Favier F, Marchal S, Azza S, Branlant G, et al (1999) Apo and holo crystal structures of an NADP-dependent aldehyde dehydrogenase from Streptococcus mutans. J Mol Biol 290: 161–173. 10388564 10.1006/jmbi.1999.2853

[3] Abdullaev ZKh, Bodrova ME, Chernyak BV, Dolgikh DA, Kluck RM, et al (2002) A cytochrome c mutant with high electron transfer and antioxidant activities but devoid of apoptogenic effect. Biochem J 362: 749–754. 11879204 10.1042/0264-6021:3620749 PMC1222441

[4] Wierenga RK, Hol WG, Misset O, Opperdoes FR (1984) Preliminary crystallographic studies of triosephosphate isomerase from the blood parasite Trypanosoma brucei brucei. J Mol Biol 178: 487–490. 6492157 10.1016/0022-2836(84)90155-4

[5] Symmons MF, Jones GH, Luisi BF (2000) A duplicated fold is the structural basis for polynucleotide phosphorylase catalytic activity, processivity, and regulation. Struct Fold Des 8: 1215–1226. 10.1016/s0969-2126(00)00521-9 11080643

[6] Ramakrishnan C, Dani S, Ramasarma TR (2002) A conformational analysis of Walker motif A [GXXXXGKT (S)] in nucleotide-binding and other proteins. Protein Eng 15: 783–798. 12468712 10.1093/protein/15.10.783

[7] Martinez-Liarte JH, Iriarte A, Martinez-Carrion M (1992) Inorganic phosphate binding and electrostatic effects in the active centre of aspartate aminotransferase apoenzyme. Biochemistry 31: 2712–2719. 1547211 10.1021/bi00125a011

[8] Meiering EM, Bycroft M, Fersht AR (1991) Characterization of phosphate binding in the active site of barnase by site-directed mutagenesis and NMR. Biochemistry 30: 11348–11356. 1958671 10.1021/bi00111a022

[9] Smith SO, Farr-Jones S, Griffin RG, Bachovchin WW (1989) Crystal versus solution structures of enzymes: NMR spectroscopy of a crystalline serine protease. Science 244: 961–964. 2499045 10.1126/science.2499045

[10] He QY, Mason AB, Nguyen V, MacGillivray RT, Woodworth RC (2000) The chloride effect is related to anion binding in determining the rate of iron release from the human transferrin N-lobe. Biochem J 350: 909–915. 10970808 PMC1221326

[11] Chakrabarti P (1993) Anion binding sites in protein structures. J Mol Biol 234: 463–482. 8230226 10.1006/jmbi.1993.1599

[12] Denessiouk KA, Rantanen VV, Johnson MS (2001) Adenine recognition: a motif present in ATP-CoA-, NAD-, NADP- and FAD-dependent proteins. Proteins Struct Funct Bioinformatics 44: 282–291. 10.1002/prot.1093 11455601

[13] Denessiouk KA, Johnson MS, Denesyuk AI (2005) Novel C^α^NN structural motif for protein recognition of phosphate ions. J Mol Biol 345: 611–629. 15581902 10.1016/j.jmb.2004.10.058

[14] Watson JD, Milner-White EJ (2002) A novel main-chain anion-binding site in proteins: the nest. A particular combination of φ, ψ values in successive residues gives rise to anion-binding sites that occur commonly and are found often at functionally important regions. J Mol Biol 315: 171–182. 11779237 10.1006/jmbi.2001.5227

[15] Milner-White EJ, Russel MJ (2005) Sites for phosphates and iron–sulfur thiolates in the first membranes: 3 to 6 residue anion-binding motifs (Nests). Origins of Life and Evolution of Biospheres 35: 19–27. 10.1007/s11084-005-4582-7 15889648

[16] Bianchi A, Giorgi C, Ruzza P, Toniolo C, Milner-White EJ (2012) A synthetic hexapeptide designed to resemble a proteinaceous P-loop nest is shown to bind inorganic phosphate. Proteins 80: 1418–1424. 22275093 10.1002/prot.24038

[17] Kinoshita K, Sadanami K, Kidera A, Go N (1999) Structural motif of phosphate binding site common to various protein superfamilies: all-against-all structural comparison of protein–mononucleotide complexes. Protein Eng 12: 11–14. 10065705 10.1093/protein/12.1.11

[18] Denesyuk AI, Denessiouk KA, Korpela T, Johnson MS (2003) Phosphate group binding “cup” of PLP-dependent and non-PLP-dependent enzymes: leitmotif and variations. Biochim Biophys Acta 1647: 234–238. 12686139 10.1016/s1570-9639(03)00057-8

[19] Remington S, Wiegand G, Huber R (1982) Crystallographic refinement and atomic models of two different forms of citrate synthase at 2.7 and 1.7 A resolution. J Mol Biol 158: 111–152. 7120407 10.1016/0022-2836(82)90452-1

[20] Denessiouk KA, Johnson MS (2003) Acceptor–donor–acceptor motifs recognize the Watson–Crick, Hoogsteen and sugar “Acceptor–donor–acceptor” edges of adenine and adenosine-containing ligands. J Mol Biol 333: 1025–1043. 14583197 10.1016/j.jmb.2003.09.017

[21] Ledvina PS, Yao N, Choudhary A, Quiocho FA (1996) Negative electrostatic surface potential of protein sites specific for anionic ligands. Proc Natl Acad Sci U S A 93: 6786–6791. 8692896 10.1073/pnas.93.13.6786 PMC39105

[22] Liu MT, Wuebbens MM, Rajagopalan KKV, Schindelin H (2000) Crystal structure of the gephyrin-related molybdenum cofactor biosynthesis protein MogA from Escherichia coli. J Biol Chem 275: 1814–1822. 10636880 10.1074/jbc.275.3.1814

[23] Richardson JS, Richardson DC (1988) Amino acid preferences for specific locations at the ends of α helices. Science 240: 1648–1652. 3381086 10.1126/science.3381086

[24] Anfinsen CB, Haber E, Sela M, White FH Jr (1961) The kinetics of formation of native ribonuclease during oxidation of the reduced polypeptide chain. Proc Natl Acad Sci U S A 47: 1309–1314. 13683522 10.1073/pnas.47.9.1309 PMC223141

[25] Sheet T, Banerjee R (2010) Sulfate ion interaction with ‘anion recognition’ short peptide motif at the N-terminus of an isolated helix: A conformational landscape. J Struct Biol 171: 345–352. 20570734 10.1016/j.jsb.2010.06.003

[26] Markovich D (2001) Physiological roles and regulation of mammalian sulfate transporters. Physiol Rev 81: 1499–1533. 11581495 10.1152/physrev.2001.81.4.1499

[27] Banerjee R, Basu G (2002) A short Aib/Ala-based peptide helix is as stable as an Ala-based peptide helix double its length. ChemBioChem 12: 1263–1266. 10.1002/1439-7633(20021202)3:12<1263::AID-CBIC1263>3.0.CO;2-O 12465037

[28] Banerjee R, Chattopadhyay S, Basu G (2009) Conformational preferences of a short Aib/Ala-based water-soluble peptide as a function of temperature. Proteins 76: 184–200. 19137603 10.1002/prot.22337

[29] Karle IL, Flippen-Anderson JL, Gurunath R, Balaram P (1994) Facile transition between 3_10_- and α-helix: structures of 8, 9 and 10 residue peptides containing the (Leu-Aib-Ala)2-Phe-Aib fragment. Protein Sci 3: 1547–1555. 7833814 10.1002/pro.5560030920 PMC2142939

[30] Crisma M, Andreetto E, De Zotti M, Moretto A, Peggion C, et al (2007) Crystal-state 3D-structural characterization of novel, Aib-based, turn and helical peptides. J Pept Sci 13: 190–205. 17226891 10.1002/psc.833

[31] Wüthrich K (1986) NMR of Proteins and Nucleic Acids. John Wiley and Sons, New York.

[32] Güntert P, Mumenthaler C, Wüthrich K (1997) Torsion angle dynamics for NMR structure calculation with the new program DYANA. J Mol Biol 273: 283–98. 9367762 10.1006/jmbi.1997.1284

[33] Guex N, Peitsch MC (1997) SWISS-MODEL and the Swiss-PdbViewer: An environment for comparative protein modeling. Electrophoresis 18: 2714–2723. 9504803 10.1002/elps.1150181505

[34] Louie GV, Brayer GD (1990) High-resolution refinement of yeast iso-1-cytochrome c and comparisons with other eukaryotic cytochromes c. J Mol Biol 214: 527–555. 2166169 10.1016/0022-2836(90)90197-T

[35] Lake MW, Wuebbens MM, Rajagopalan KV, Schindelin H (2001) Mechanism of ubiquitin activation revealed by the structure of a bacterial MoeB-MoaD complex. Nature 414: 325–329. 11713534 10.1038/35104586

[36] Accelrys Software Inc., Discovery Studio Modeling Environment, (2010) Release 2.5.5, San Diego: Accelrys Software Inc.

[37] Kabsch W, Sander C (1983) Dictionary of protein secondary structure: pattern recognition of hydrogen-bonded and geometrical features. Biopolymers 22: 2577–2637. 6667333 10.1002/bip.360221211

[38] Morris GM, Huey R, Lindstrom W, Sanner MF, Belew RK, et al (2009) AutoDock4 and AutoDockTools4: Automated docking with selective receptor flexibility. J Comput Chem 30: 2785–2791. 19399780 10.1002/jcc.21256 PMC2760638

[39] Morris GM, Goodsell DS, Halliday RS, Huey R, Hart WE, et al (1998) Automated docking using a Lamarckian genetic algorithm and an empirical binding free energy function. J Comput Chem 19: 1639–1662.

[40] Holt PA, Chaires JB, Trent JO (2008) Molecular docking of intercalators and groove-binders to nucleic acids using Autodock and Surflex. J Med Chem 51: 3878–3894. 18642866 10.1021/ci800063v PMC2755229

[41] Brandl M, Weiss MS, Jabs A, Sühnel J, Hilgenfeld R (2001) C-H…π-interactions in proteins. J Mol Biol 307: 357–377. 11243825 10.1006/jmbi.2000.4473

[42] Derewenda ZS, Lee L, Derewenda U (1995) The occurrence of C-H…O hydrogen bonds in proteins. J Mol Biol 252: 248–262. 7674305 10.1006/jmbi.1995.0492

[43] Huey R, Morris GM, Olson AJ, Goodsell DS (2007) A semiempirical free energy force field with charge-based desolvation. J Comput Chem 28: 1145–1152. 17274016 10.1002/jcc.20634

[44] Hol WGJ, van Duijnen PT, Berendsen HJC (1978) The α-helix dipole and the properties of proteins. Nature 273: 443–446. 661956 10.1038/273443a0

[45] Aquist J, Luecke H, Quiocho FA, Warshel A (1991) Dipoles localized at helix termini of proteins stabilize charges. Proc Natl Acad Sci USA 88: 2026–2030. 2000410 10.1073/pnas.88.5.2026 PMC51159

[46] Ledvina PS, Tsai AL, Wang Z, Koehl E, Quiocho FA (1998) Dominant role of local dipolar interactions in phosphate binding to a receptor cleft with an electrostatic charge surface: equilibrium, kinetic and crystallographic studies. Protein Sci 7: 2550–2559. 9865949 10.1002/pro.5560071208 PMC2143890

[47] He JJ, Quiocho FA (1993) Dominant role of local dipoles in stabilizing uncompensated charges on a sulfate sequestered in a periplasmic active transport protein. Protein Sci 2: 1643–1647. 8251939 10.1002/pro.5560021010 PMC2142251

[48] Demuth C, Zerbe O, Rognan D, Soll R, Beck-Sickinger A, et al (2001) A rationally designed oligopeptide shows significant conformational changes upon binding to sulphate ions. Biosensors & Bioelectronics 16: 783–789. 11679256 10.1016/s0956-5663(01)00221-4

[49] Sansom MS, Adcock C, Smith GR (1998) Modelling and simulation of ion channels: applications to the nicotinic acetylcholine receptor. J Struct Biol 121: 246–262. 9615441 10.1006/jsbi.1997.3950

[50] Allen TW, Andersen OS, Roux B (2004) Energetics of ion conduction through the gramicidin channel. Proc Natl Acad Sci U S A 101: 117–222. 14691245 10.1073/pnas.2635314100 PMC314148

[51] Aburi M, Smith PE (2004) A combined simulation and Kirkwood–Buff approach to quantify cosolvent effects on the conformational preferences of peptides in solution. J Phys Chem B 108: 7382–7388.

[52] Pettersen EF, Goddard TD, Huang CC, Couch GS, Greenblatt DM, et al (2004) UCSF Chimera–a visualization system for exploratory research and analysis. J Comput Chem 25: 1605–1612. 15264254 10.1002/jcc.20084

[53] Hess B, Kutzner C, van der Spoel D, Lindahl E (2008) GROMACS 4: Algorithms for highly efficient, load-balanced, and scalable molecular simulation. J Chem Theory Comput 4: 435–447. 26620784 10.1021/ct700301q

[54] van der Spoel D, Lindahl E, Hess B, Groenhof G, Mark AE, et al (2005) GROMACS: Fast, flexible, and free. J Comput Chem 26: 1701–1719. 16211538 10.1002/jcc.20291

[55] Jorgensen WL, Tirado-Rives J (1988) The OPLS [optimized potentials for liquid simulations] potential functions for proteins, energy minimizations for crystals of cyclic peptides and crambin. J Am Chem Soc 110: 1657–1666. 27557051 10.1021/ja00214a001

[56] Dennington R, Keith T, Millam J (2009) GaussView, Version 5.0.8.: *Semichem Inc.*, Shawnee Mission KS.

[57] Frisch MJ, Trucks GW, Schlegel HB, Scuseria GE, Robb MA, et al.. (2009) Gaussian 09, Gaussian, Inc., Wallingford CT, Revision A.02.

[58] Berendsen HJC, Grigera JR, Straatsma TP (1987) The missing term in effective pair potentials. J Phys Chem 91: 6269–6271.

[59] Berendsen HJC, Postma JPM, van Gunsteren WF, DiNola A, Haak JR (1984) Molecular dynamics with coupling to an external bath. J Chem Phys 81: 3684–3690.

[60] Hess B, Bekker H, Berendsen HJC, Fraaije JGEM (1997) LINCS: A linear constraint solver for molecular simulations. J Comput Chem 18: 1463–1472.

[61] Darden T, York D, Pedersen L (1993) Particle mesh Ewald: An N-log(N) method for Ewald sums in large systems. J Chem Phys 98: 10089–10092.

