# Supplementary figures and images for: Conformational Preference of ‘CαNN’ Short Peptide Motif towards Recognition of Anions
Source: PLoS One. 2013 Mar 13;8(3):e57366. doi: 10.1371/journal.pone.0057366 (PMC3596363; doi:10.1371/journal.pone.0057366)

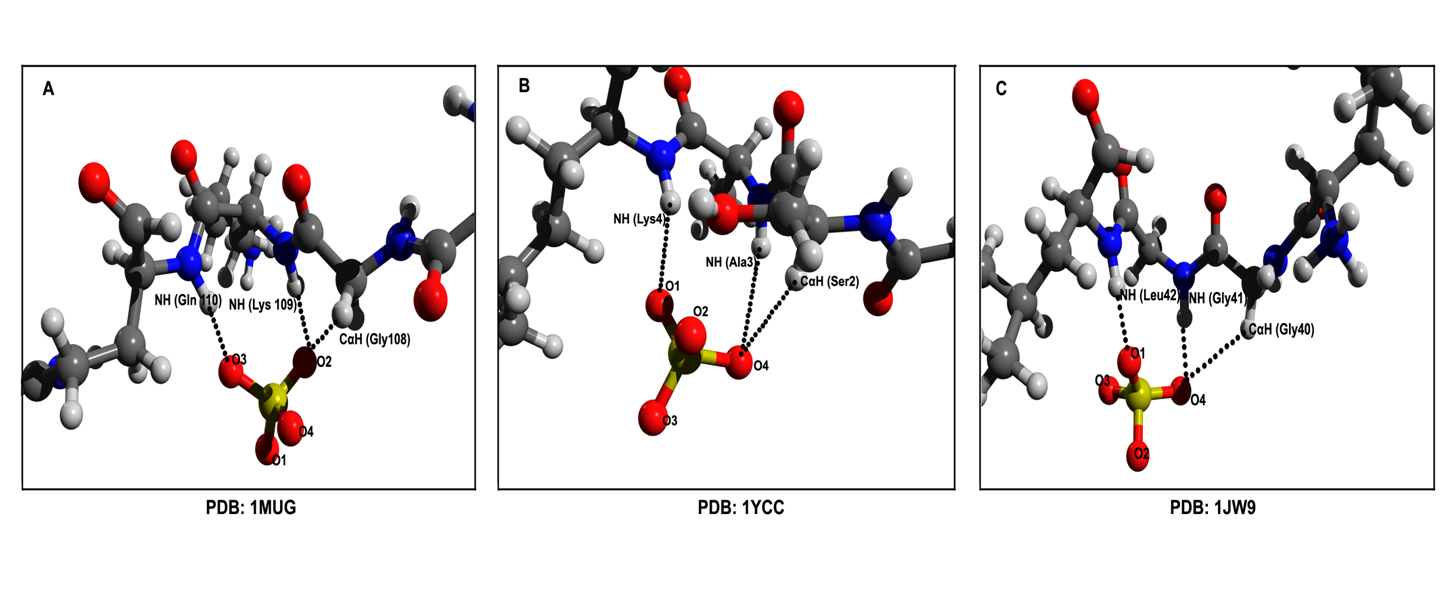

Supplement: Figure S2 — Representation of interaction of sulfate ion with the ‘CαNN’ segment of the respective proteins found in the crystal structure deposited in PDB. (TIF) [file pone.0057366.s002.tif]

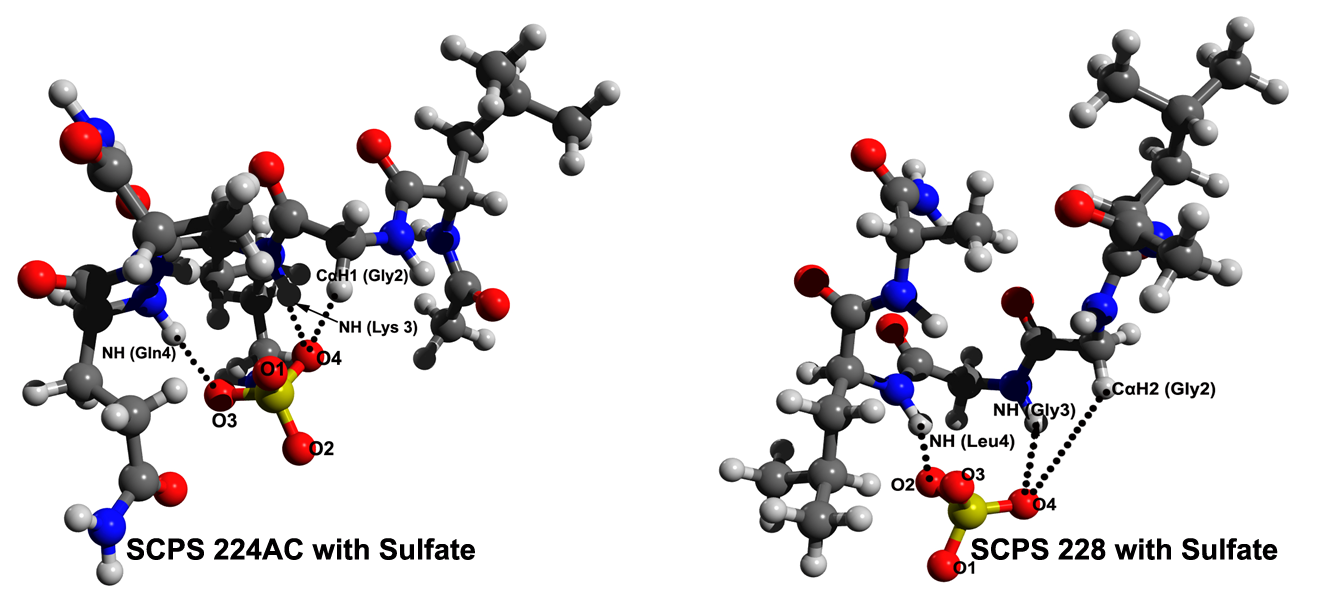

Supplement: Figure S3 — Representation of sulfate ion interactions with the ‘experimental NMR structure’ of SCPS224Ac and the ‘native model structure’ of SCPS228 showing that the ‘CαNN’ motif segment can recognize sulfate ion even in short sequences. (TIF) [file pone.0057366.s003.tif]

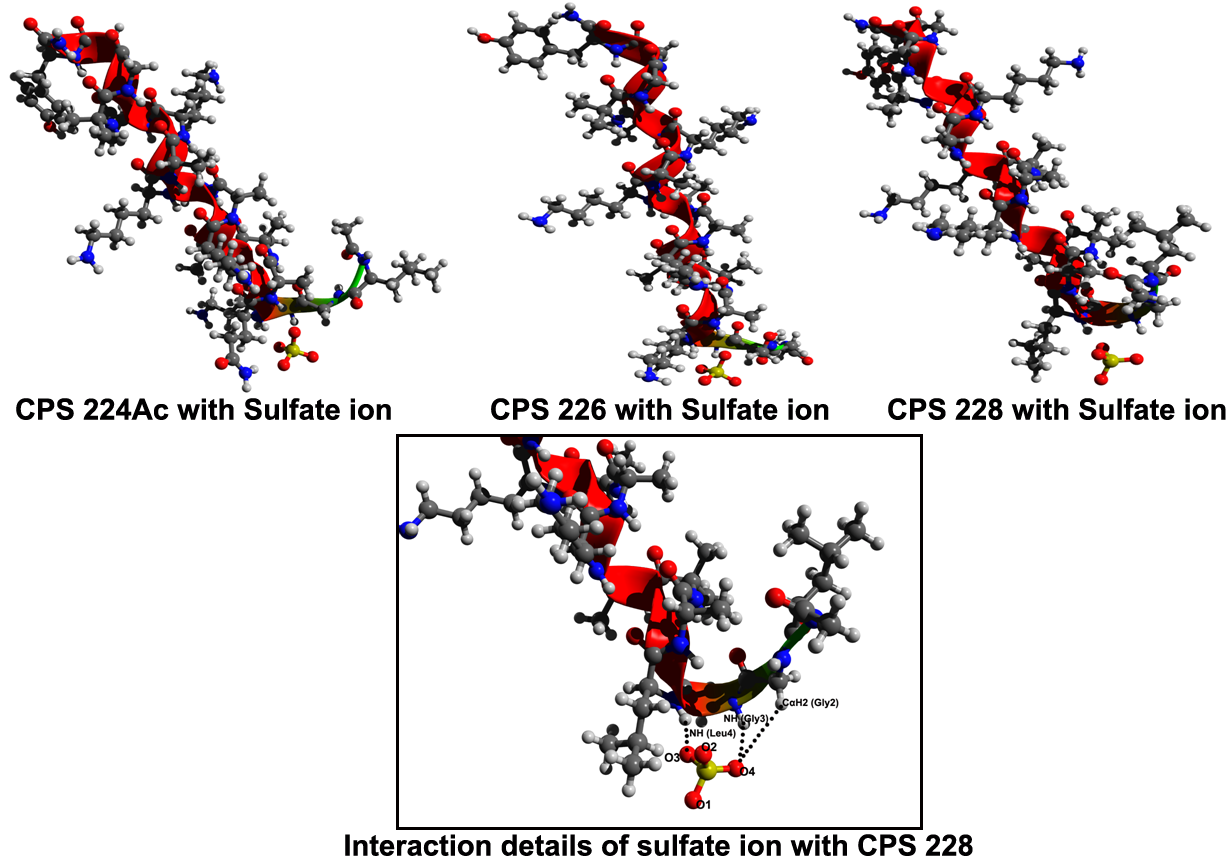

Supplement: Figure S4 — Representation of sulfate ion interactions with the ‘native model’ structure of CPS224Ac, CPS226 and CPS228 showing that the ‘CαNN’ motif segment at the N-terminus act as the only recognition site for sulfate ion in each case. (TIF) [file pone.0057366.s004.tif]

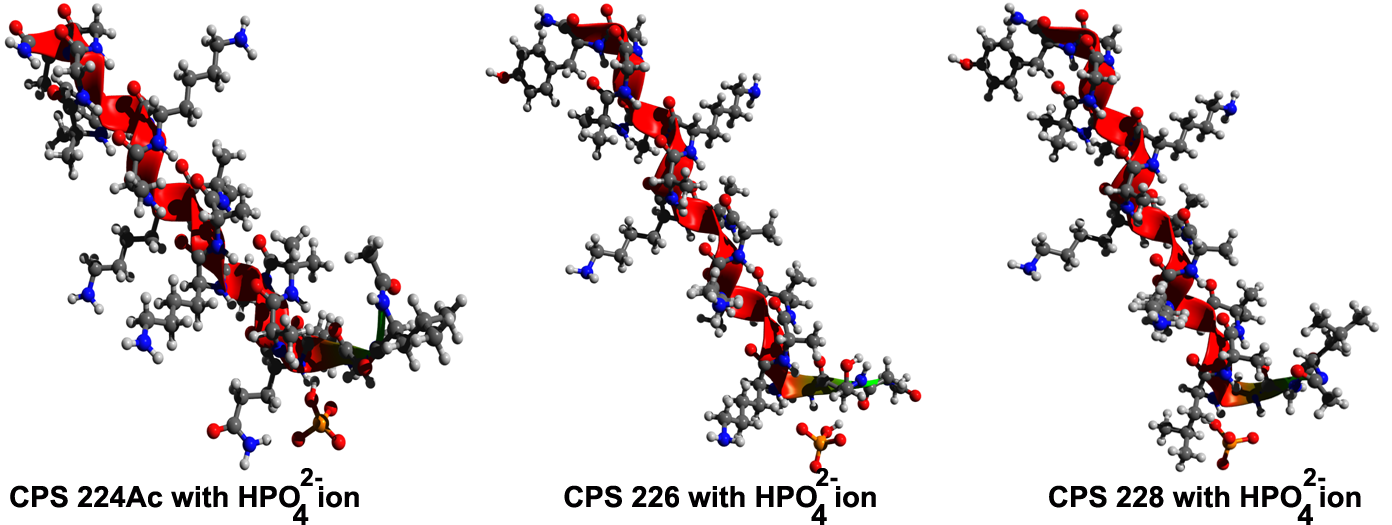

Supplement: Figure S5 — Representation of phosphate ion interactions with the ‘native model’ structure of CPS224Ac, CPS226 and CPS228 showing that the ‘CαNN’ motif segment at the N-terminus act as the only recognition site for phosphate ion in each case. (TIF) [file pone.0057366.s005.tif]

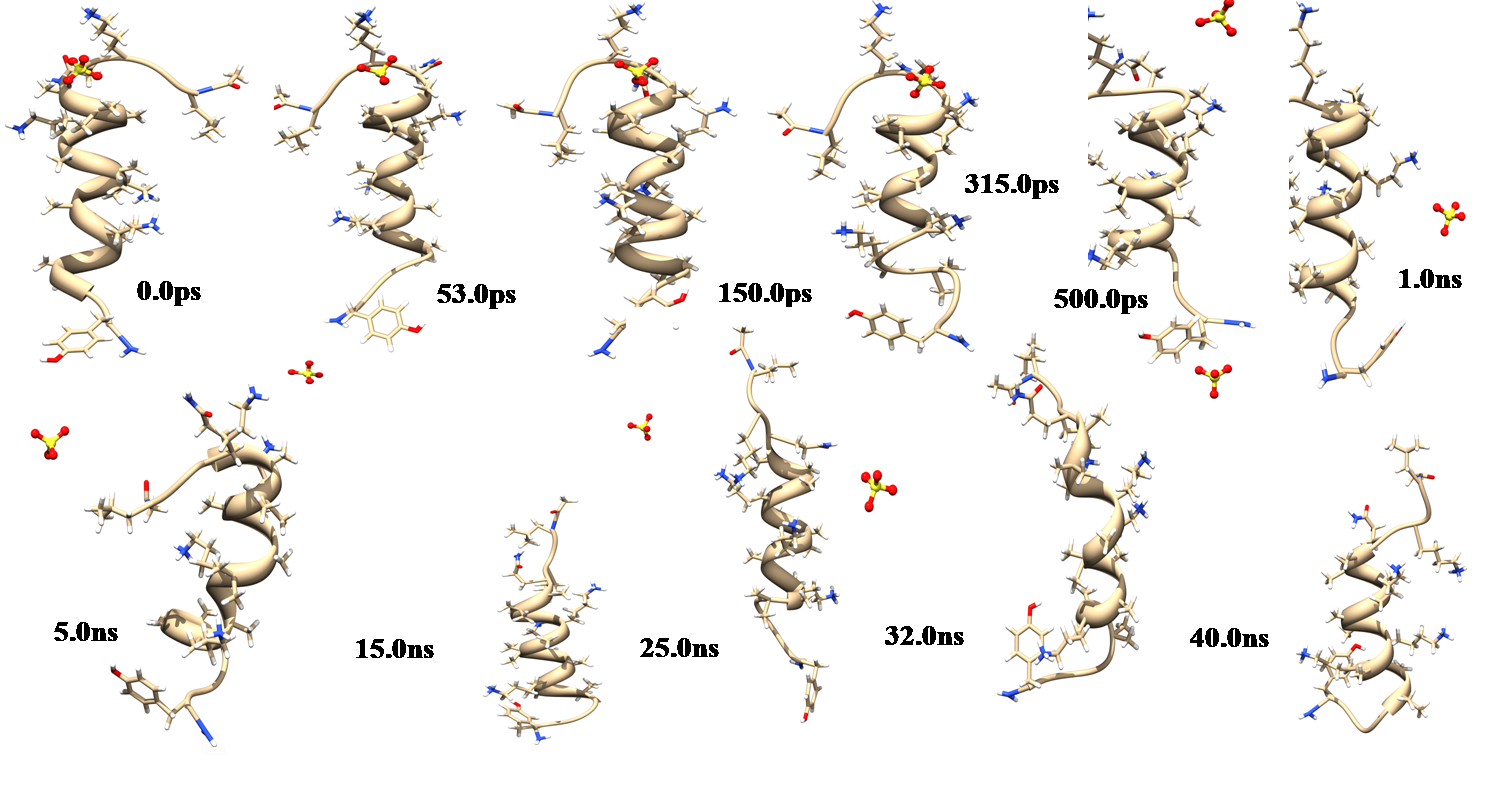

Supplement: Figure S6 — A few snapshots of MD trajectories (40 ns) showing the interaction of sulfate ion with the ‘CαNN’ motif in ‘Experimental NMR’ structure of CPS224Ac. (TIF) [file pone.0057366.s006.tif]

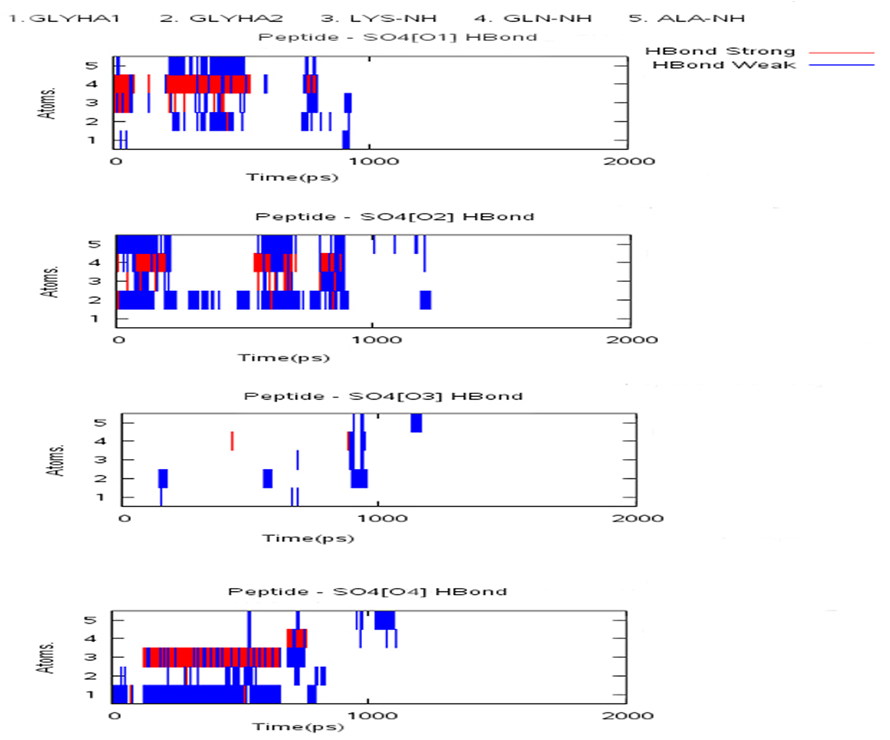

Supplement: Figure S7 — Partial representation of sulfate ion interaction with the CαNN′ motif segment of CPS224Ac (‘Native model’ structure) in Molecular Dynamics experiment at 276K (blue lines indicate weak interaction while red lines indicate strong H-bond), showing out of four oxygen two are simultaneously interacting with constituent main-chain atoms through H-bond. (TIF) [file pone.0057366.s007.tif]

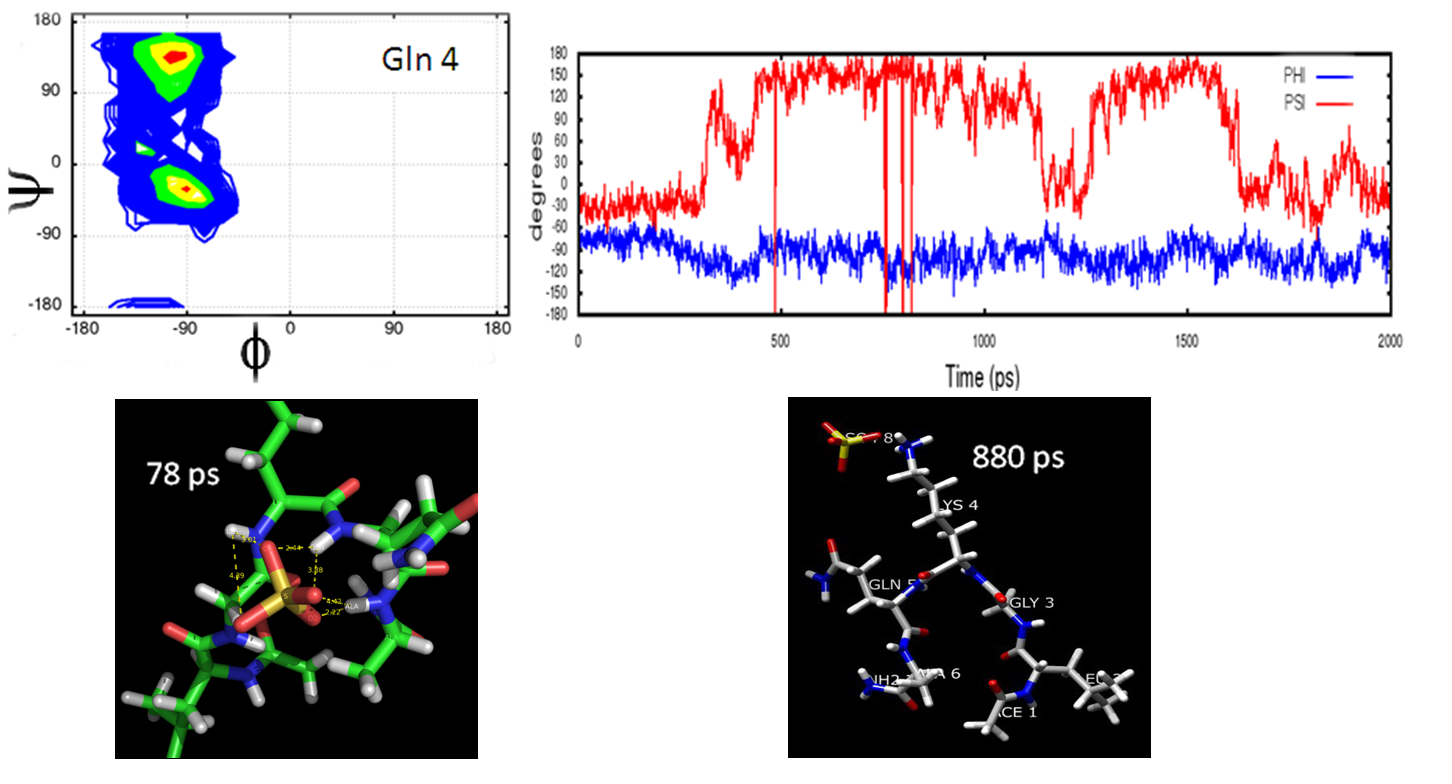

Supplement: Figure S8 — Distribution of backbone dihedral angles (φ, ψ) of the Gln4 residue during the MD simulation of sulfate ion interactions with the ‘experimental NMR structure’ of SCPS224Ac emphasizing its existence in helical conformation during interaction of sulfate ion with ‘CαNN’ motif segment peptide and the role of Aib6 residue pertaining the non-canonical helical conformation of Gln4 in CPS224Ac in the absence of sulfate ion. Two snapshots of the interaction are shown when the sulfate ion is close to ‘CαNN’ segment and apart from the segment. (TIF) [file pone.0057366.s008.tif]
