# Supplementary material for: Conformational Preference of ‘CαNN’ Short Peptide Motif towards Recognition of Anions
Source: PLoS One. 2013 Mar 13;8(3):e57366. doi: 10.1371/journal.pone.0057366 (PMC3596363; doi:10.1371/journal.pone.0057366)
Supplement: Table S1 — Representation of interaction parameters ((X)H…O Distance (Å) and <X-H…O Angle (°)) of anion (sulfate ion) with ‘CαNN’ segment obtained from the respective crystal structures of proteins reported in PDB (aBarrett etal.,1998; bLouie and Brayer 1990; cLake etal., 2001). (DOC) [file pone.0057366.s009.doc]

| **PDB ID** | **Ligand** | **(X)H…O Distance (Å)** | | | **X-H…O Angle (°)** | | |
| --- | --- | --- | --- | --- | --- | --- | --- |
|  |  | **C^α^_-1_** | **N_0_** | **N_+1_** | **C^α^_-1_** | **N_0_** | **N_+1_** |
| 1MUG^a^ | SO_4_^2-^ | 2.37 | 1.79 | 1.74 | 135.97 | 156.82 | 167.77 |
| 1YCC^b^ | SO_4_^2-^ | 2.67 | 2.59 | 2.3 | 160.72 | 156.01 | 141.06 |
| 1JW9^c^ | SO_4_^2-^ | 2.89 | 1.84 | 1.77 | 132.28 | 174.39 | 179.27 |

**Table S1:** Representation of interaction parameters ((X)H…O Distance (Å) and X−H…O Angle (°)) of anion (sulfate ion) with ‘C^α^NN’ segment obtained from the respective crystal structures of proteins reported in PDB (^a^Barrett etal.,1998; ^b^Louie and Brayer 1990; ^c^Lake etal., 2001)
