# Supplementary material for: Conformational Preference of ‘CαNN’ Short Peptide Motif towards Recognition of Anions
Source: PLoS One. 2013 Mar 13;8(3):e57366. doi: 10.1371/journal.pone.0057366 (PMC3596363; doi:10.1371/journal.pone.0057366)
Supplement: Table S2 — Interaction parameters between the sulfate/phosphate ion with the related ‘CαNN’ segment of the short 5-residue peptides (truncated version of the 18-residue sequences) in a context free system (250 docked structures of the individual conformation) are described in terms of X-H—O (where X = Cα−1/N0/N+1) distances (Å) and angles (°) indicating the nature of H-bond formation (mean value of the parameters in parenthesis). The estimated binding free energy gives a relative affinity for anion which depends on the conformational status of the ‘CαNN’ segment. (DOC) [file pone.0057366.s010.doc]

| **Peptides** | **Confor-mations**  **at C^α^NN segment** | **Sulfate ion** | | | | | | | **Phosphate ion** | | | | | | |
| --- | --- | --- | --- | --- | --- | --- | --- | --- | --- | --- | --- | --- | --- | --- | --- |
|  |  | **(X)H…O Distance (Å)** | | | **X-H…O Angle (°)** | | | **Binding free energy (Kcal/Mol)** | **(X)H…O Distance (Å)** | | | **X-H…O Angle (°)** | | | **Binding free energy (Kcal/Mol)** |
|  |  | **C ^α^_-1_** | **N_0_** | **N_+1_** | **C^α^_-1_** | **N_0_** | **N_+1_** |  | **C^α^_-1_** | **N_0_** | **N_+1_** | **C^α^_-1_** | **N_0_** | **N_+1_** |  |
| **SCPS224Ac** | **NMR** | **3.51-3.57 (3.54)** | **1.79-1.82 (1.81)** | **1.94-2.14 (2.01)** | **100.59-104.71 (102.004)** | **152.88-156.01 (154.74)** | **165.07-167.66 (166.27)** | **-3.86** | **3.49-3.76 (3.597)** | **1.84-2.1 (1.90)** | **1.67-2.19 (1.97)** | **98.73-105.87 (101.1)** | **158.65-165.79 (161.73)** | **167.25-171.87 (170.64)** | **-3.12** |
|  | **Native** | **3.06-3.14 (3.09)** | **2.41-2.47 (2.42)** | **1.65-1.85 (1.69)** | **105.32-106.31 (105.96)** | **98.49-103.12 (99.47)** | **173.04-175.2 (173.57)** | **-3.26** | **3.37-3.42 (3.41)** | **2.37-2.46 (2.45)** | **1.71-1.73 (1.71)** | **110.63-111.05 (110.76)** | **100.93-101.51 (101.00)** | **173.21-176.18 (175.84)** | **-2.35** |
|  | **Extended** | **5.8-6.00 (5.87)** | **4.45-4.56 (4.48)** | **2.1-2.15 (2.12)** | **86.65-89.73 (87.63)** | **50-62.2 (53.45)** | **131.46-142.9 (141.85)** | **-1.82** | **3.5-3.76 (3.55)** | **2.1-2.18 (2.16)** | **4.3-4.72 (4.57)** | **110.46-114.91 (112.53)** | **141.45-145.78 (143.03)** | **33.91-43.97 (40.48)** | **-1.48** |
|  |  |  |  |  |  |  |  |  |  |  |  |  |  |  |  |
| **SCPS226** | **Native** | **2.79-3.01 (2.87)** | **2.60-2.72 (2.65)** | **1.63-1.74 (1.66)** | **108.47-110.97 (109.7)** | **96.74-98.78 (97.31)** | **173.29-177.78 (176.12)** | **-3.06** | **2.23-2.47 (2.4)** | **1.77-2.17 (1.89)** | **1.95-2.1 (1.99)** | **127.66-141.35 (137.93)** | **151.61-164.95 (161.86)** | **166.42-170.17 (167.65)** | **-2.27** |
|  | **Extended** | **2.75-3.11 (3.08)** | **1.98-2.14 (2.12)** | **5.7-5.89 (5.82)** | **117.37-117.80 (117.7)** | **140.88-149.53 (146.65)** | **36.05-39.85 (38.12)** | **-1.74** | **3.35-3.59 (3.53)** | **2.14-2.29 (2.25)** | **1.98-2.21 (2.1)** | **113.38-114.92 (114.36)** | **144.04-146.88 (145.46)** | **145.19-148.06 (146.57)** | **-1.27** |
|  |  |  |  |  |  |  |  |  |  |  |  |  |  |  |  |
| **SCPS228** | **Native** | **3.66-3.88 (3.77)** | **1.89-2.15 (2.02)** | **1.78-2.08 (1.83)** | **119.91-124.19 (122.87)** | **136.13-144.49 (142.17)** | **170.07-178.6 (176.41)** | **-2.20** | **3.59-4.04 (3.82)** | **1.77-2.14 (1.95)** | **1.69-2.18 (1.84)** | **112.42-124.41 (117.85)** | **134.32-153.9 (139.78)** | **150.54-173.15 (165.85)** | **-1.32** |
|  | **Extended** | **3.31-3.5 (3.36)** | **1.74-1.8 (1.76)** | **5.14-5.3 (5.26)** | **96.7-99.72 (98.02)** | **173.04-178.25 (176.13)** | **44.17-50.36 (47.8)** | **-1.53** | **3.42-3.77 (3.66)** | **1.88-2.13 (2.07)** | **5.35-5.59 (5.48)** | **95.22-104.8 (100.13)** | **165.28-175.8 (172.75)** | **42.35-49.95 (46.16)** | **-1.24** |

**Table S2:** Interaction parameters between the sulfate / phosphate ion with the related ‘C^α^NN’ segment of the short 5-residue peptides (truncated version of the 18-residue sequences) in a context free system (250 docked structures of the individual conformation) are described in terms of X−H---O (where X= C^α^_-1_/N_0_/N_+1_) distances (Å) and angles (°) indicating the nature of H-bond formation (mean value of the parameters in parenthesis). The estimated binding free energy gives a relative affinity for anion which depends on the conformational status of the ‘C^α^NN’ segme
